# SCR-6852, an oral and highly brain-penetrating estrogen receptor degrader (SERD), effectively shrinks tumors both in intracranial and subcutaneous ER + breast cancer models

**DOI:** 10.1186/s13058-023-01695-4

**Published:** 2023-08-14

**Authors:** Feng Zhou, Guimei Yang, Liting Xue, Yajing Liu, Yao Guo, Ji Zhu, Linlin Yuan, Peng Gu, Feng Tang, Jinwen Shan, Renhong Tang

**Affiliations:** 1 State Key Laboratory of Neurology and Oncology Drug Development, Nanjing, China; 2 Simcere Zaiming Pharmaceutical Co., Ltd., Shanghai, China; 3 Jiangsu Simcere Pharmaceutical Co., Ltd., Nanjing, China

**Keywords:** SCR-6852, SERD, Fulvestrant, Estrogen receptor, Breast cancer, Breast cancer brain metastasis

## Abstract

**Background:**

Targeted estrogen receptor degradation has been approved to effectively treat ER + breast cancers. Due to the poor bioavailability of fulvestrant, the first generation of SERD, many efforts were made to develop oral SERDs. With the approval of Elacestrant, oral SERDs demonstrated superior efficacy than fulvestrant. However, due to the poor ability of known SERDs to penetrate the blood–brain barrier (BBB), breast cancer patients with brain metastasis cannot benefit from clinical SERDs.

**Methods:**

The ER inhibitory effects were evaluated on ERα protein degradation, and target genes downregulation. And anti-proliferation activities were further determined in a panel of ER + breast cancer cell lines*.* The subcutaneous and intracranial ER + tumor models were used to evaluate the efficacy of anti-tumor effects. Brain penetrability was determined in multiple animal species.

**Results:**

SCR-6852 is a novel SERD and currently is under early clinical evaluation. In vitro studies demonstrated that it strongly induced both wildtype and mutant ERα degradation. It potently inhibited cell proliferation in a panel of ER + breast cancer cell lines, including the cell lines containing *ESR1* mutations (Y537 and D538). Furthermore, SCR-6852 exhibited pure antagonistic activities on the ERɑ signal axis identified both in vitro and in vivo. Oral administration of SCR-6852 at 10 mg/kg resulted in tumor shrinkage which was superior to Fulvestrant at 250 mg/kg, notably, in the intracranial tumor model, SCR-6852 effectively inhibited tumor growth and significantly prolonged mice survival, which correlated well with the high exposure in brains. In addition to mice, SCR-6852 also exhibited high brain penetrability in rats and dogs.

**Conclusions:**

SCR-6852 is a novel SERD with high potency in inducing ERα protein degradation and pure antagonistic activity on ERɑ signaling in vitro and in vivo. Due to the high brain penetrability, SCR-6852 could be used to treat breast patients with brain metastasis.

**Supplementary Information:**

The online version contains supplementary material available at 10.1186/s13058-023-01695-4.

## Background

Breast cancer (BC) is the most common malignancy in women around the world, and the overall rates of BC incidence and mortality for the world population have continuously increased [1, 2]. According to the GLOBOCAN 2020 estimation by the International Agency for Research on Cancer, female breast cancer had surpassed lung cancer as the most commonly diagnosed cancer, with an estimated 2.3 million new cases per year [3]. Approximately 80% of breast cancers are estrogen receptor-positive (ER +) which results in the most breast cancer deaths [4, 5]. Despite substantial improvements achieved both in disease-free survival and overall survival with endocrine therapies in early-stage breast cancers [6], up to 30% of patients diagnosed with operable ER + tumors eventually metastasized [7].

Estrogen receptor has been targeted for breast cancer treatment for over a century [8, 9]. Endocrine therapy is the main therapeutic choice in clinical practices for ER-positive metastatic breast cancer (mBC) patients with proven clinical benefits [10]. Tamoxifen, the first ER modulator has reduced breast cancer recurrence and annual mortality rate by 50% and 31%, respectively, since the approval by the Food and Drug Administration (FDA) for the treatment of women with advanced breast cancer [11]. However, numerous studies demonstrated that tamoxifen is a partial ER agonist with both additive and antagonistic effects to estradiol [12]. Furthermore, some resistant mutations (e.g. Y537S and D538G) are observed in the ER ligand-binding domain (LBD) after the long-term treatment of tamoxifen, which leads to the disease progression [13]. Estrogen receptor degrader/down-regulator works as a pure ER antagonist that acts by binding to ER and consequently induces the rapid degradation of ER [8, 14]. Fulvestrant is the first SERD available in clinical practice [15] and the efficacies were well demonstrated by extensive clinical trials. In a second-line setting trial, Fulvestrant was still proved to be effective in patients having experienced progression after previous endocrine therapy with tamoxifen, with clinical benefits both in PFS (6.5 vs. 5.5 months; *p* = 0.05) and OS (26.4 months with Fulvestrant HD 500 mg and 22.3 months with 250 mg dose regimen *p* = 0.05) [16]. Clinical investigation revealed that side effects associated with tamoxifen was not observed in Fulvestrant either in monotherapy or in combination with other agents, for the treatment of ER-positive advanced breast cancer [17, 18]. However, poor bioavailability, slow action associated with intramuscular injection, and low response, these limitations of Fulvestrant have driven a critical need to develop a clinically proven, orally bioavailable SERD. At present, a few numbers of new generated oral SERDs were developed including Elacestrant (RAD-1901) which was recently approved for medical use in the United States [19], GDC-9545 (Roche) and AZD9833 (AstraZeneca). The last two oral SERDs also demonstrated promising antitumor activity in patients with advanced ER-positive breast cancer in phase 2 trials [20–22].

Breast cancer brain metastasis (BCBM) is the second most common cause of brain metastasis, and its occurrence has been rising in the past two decades with significant improvement in the survival of advanced breast cancer patients [23]. The current treatment options for ER-positive breast cancer patients with brain metastases are limited, including surgical resection and local radiotherapy. However, not all patients are suitable for those treatments. And patients with brain metastases who receive these treatments eventually develop refraction in a short time. Medicines with high BBB penetration capability could be a better choice for those brain metastasis patients. However, although several SERDs were investigated in the clinic, few SERDs were reported with brain penetration character and had been investigated in brain MBC patients, with the exception of OP-1250 from Olema Oncology. Thus, the development of an oral SERD with high BBB penetrability is still an unmet medical need. SCR-6852 is an oral, highly BBB penetrable and selective ERα degrader which is under clinical evaluation. Preclinical data showed that SCR-6852 strongly inhibited the growth of ER-positive breast cancer cells by inducing ERα degradation and following the complete inhibition of ER target genes transcription. In ER + subcutaneous tumors, SCR-6852 demonstrated superior anti-tumor activities than Fulvestrant. Notably, SCR-6852 possessed high BBB penetrability in multiple pre-clinical animals and significantly promotes mouse survival in an intracranial tumor model. A combination of SCR-6852 and a CDK4/6 inhibitor revealed synergistically anti-tumor activities both in vitro and in vivo.

## Methods

### Cell lines

MCF-7 (#HTB-22), CAMA-1 (#HTB-21), HCC1500 (#CRL-2329), BT-474 (#HTB-20), and SK-BR-3 (#HTB-30) cells were purchased from ATCC. Especially, MCF-7 (#86012803) used in subcutaneous xenograft mouse model was purchased from ECACC. EFM-19 (#CBP60363) was from Cobioer Bioscience. T47D (#KC-0199) was from KYinno Biotechnology. All cell authentication was conducted through short tandem repeat (STR) DNA profiling by Biowing and the routine screening for mycoplasma contamination was done using Lonza Mycoalert and Stratagene Mycosensor. Unless otherwise indicated, tissue culture supplements and medium were purchased from Hyclone, Corning, or Invitrogen. Cells were maintained as recommended by the vendor. MCF-7 was maintained in DMEM with 10% FBS, 0.01 mg/ml of Human insulin (Yeasen), and 1% NEAA. CAMA-1 was maintained in EMEM with 10% FBS. HCC1500 and EFM-19 were maintained in RPMI-1640 with 10% FBS. T47D was maintained in RPMI-1640 with 10% HI-FBS and 2 units/mL bovine insulin (Solarbio). BT-474 was maintained in Hybri-Care Medium (ATCC) with 1.5 g/L sodium bicarbonate and 10%FBS. SK-BR-3 was maintained in McCoy's 5A Medium with 10% FBS. MCF7 cells expressing the ER. Y537S variant (#CBP60380DR-3) was from Cobioer Bioscience and maintained in MEM with 10%FBS containing 1%NEAA and 1 mM NaP. The MCF7 cells harbo D538G variant (WUXI_MCF7_ER_D538G_KI) were provided by Wuxi AppTec was cultured in EMEM with 10% FBS and 1% NEAA.

### Compounds

SCR-6139, SCR-6515, and SCR-6852 were synthesized as described in (WO2021228210), GDC-9545 was synthesized described in (WO2019245974) (Compound A). AZD9833 was made as described in WO2018077630 (example 17), AZD9496 was purchased from Selleck (#S8372). ARV-471 was synthesized as described in WO2022132652 (compound 1c). Fulvestrant, 4-OH-tamoxifen, Palbociclib, Alpelisib and Elacestrant (RAD1901) were purchased from MCE.

### Animals

All experimental procedures involving animals and their care were conducted in conformity with the State Council Regulations for Laboratory Animal Management (Enacted in 1988) and were approved by the Institutional Animal Care and Use Committee of the WuXi AppTec and Simcere, People's Republic of China. Female Balb/c nude mice at 6–8 weeks of age were purchased from Shanghai Lingchang Biotechnology Co., Ltd. (Shanghai, China). Female NOD.Cg-Prkdc^scid^ Il2rg^tm1Vst^/Vst (NPG) mice at 6–8 weeks of age were purchased from Beijing Vitalstar Biotechnology Co., Ltd. (Beijing, China). Female Sprague–Dawley (SD) rats at 3 weeks of age were purchased from Charles River (Beijing, China).

### Molecular docking and molecular dynamic simulation

*Molecular docking.* The protein data bank (www.rcsb.org, PDB: 6ZOQ, resolution: 2.34) was used to derive the crystal structure of the human estrogen receptor alpha (ERα) protein. The downloaded protein was generated in the docking software MOE. The QuickPrep module of Molecular Operating Environment (MOE) 2022 is used to process the protein comprised by applying gas tier charges through the MMFF94x forcefield, adding hydrogen atoms, removing water molecules, 3D protonation of the structure, and minimizing the protein structure to a chosen gradient. Meanwhile, the WASH module and Energy Minimize of MOE were used for hydrogenation and energy minimization of small molecules.

After protein preparation and ligands preparation, 4-hydroxytamoxifen, Fulvestrant, SCR6139, and SCR6515 were docked with ERα. The active site was selected using rectangular coordinates based on the co-crystal ligand QNE. The docking algorithm was configured to use the triangle matcher placement approach [24], and the default GBVI/WSA dG technique was used as a docking function in MOE [25]. The binding poses of the compound with the highest five docking scores can be visualized in MOE. Finally, the most suitable pose was picked for the study.

### Proliferation assays

Trypsinized cells were dispensed into 384-well plates in culture media and after overnight incubation cells were treated with compounds for 7 days. Cell viability was assessed using CellTiter-Glo (Promega) according to the manufacturer’s protocol and relative luminescence units (RLU) were measured using an Envision Multilabel Reader (Perkin Elmer). Inhibition% was normalized to untreated samples and Fulvestrant treated samples or media samples. IC_50_ and Imax were analyzed with IDBS XLfit.

### In-cell Western assay

MCF-7 wild type and Y537S mutant cells were seeded at a density of 5000 cells per well into flat clear bottom tissue cultured-treated 384-well plates (Greiner) in culture media and after overnight incubation cells were treated with compounds for 24 h. Then plates were fixed with 4% formaldehyde, permeabilized with ice-cold methanol, and blocked with Odyssey Blocking Buffer (LI-COR). The fixed cells were incubated with rabbit anti- ERα antibody (CST#8644S) and mouse anti-GAPDH antibody (Abcam #ab8245), washed and stained with IRDye 800 Goat anti-Rabbit IgG (Licor#926-32211) and IRDye 680 Goat anti-Mouse IgG (Licor#926-68070). ERα levels were quantitated using Sapphire RGBNIR (Azure), the acumen eX3 imaging system. Inhibition% was normalized to untreated samples and Fulvestrant-treated samples. IC_50_ and Emax were analyzed with IDBS XLfit.

EFM-19, CAMA-1, HCC1500, T47D, and BT-474 were seeded into flat clear bottom tissue cultured-treated 96-well plates (Greiner) in culture media and after overnight incubation cells were treated with 100 nM compounds for 24 h. Then plates were fixed with 4% formaldehyde, permeabilized with ice-cold methanol, and blocked with Odyssey Blocking Buffer. The fixed cells were incubated with rabbit anti- ERα antibody and mouse anti-GAPDH antibody, washed and stained with IRDye 800 Goat anti-Rabbit IgG and IRDye 680 Goat anti-Mouse IgG. ERα levels were quantitated using Sapphire RGBNIR Inhibition% was normalized to untreated samples and Fulvestrant-treated samples.

### Western blot

The cells of MCF-7 and MCF7 ESR1Y537S were seeded in 6 well plates for 24 h. Then compounds with different concentrations were added to cell plates. After 5 days, all cell samples were lysed with ice-cold RIPA buffer (Sigma) for 30 min. The lysate supernatant was centrifuged at 4 °C centrifuge at 10000 rpm for 10 min. Protein concentrations were determined with Pierce™ BCA Protein Assay Kit (Thermo Fisher Scientific). Each sample with equal total proteins was mixed with a loading buffer. Proteins from cell lysates were separated electrophoretically using NuPAGE 4–12% Bis–Tris Gels (Thermo Fisher Scientific) in MOPS buffer (Thermo Fisher Scientific). Gels were then electroblotted onto PVDF membranes (Bio-RAD). The blots were blocked with 5% BSA (Sigma) in TBST buffer for 1 h and then incubated with primary antibodies overnight at 4 °C. Blots were washed with TBST, incubated with secondary antibodies for 1 h, and washed with TBST. The blots were detected by SuperSignal™ West Dura Extended Duration Substrate (Thermo Fisher Scientific) and scanned using Sapphire RGBNIR (Azure). The protein levels were quantitated using ImageJ and normalized to β-Actin. Percent objective protein was defined as normalized treated sample/normalized untreated cells × 100. Primary antibodies: Rabbit anti-ERα(D8H8) (1:3000, CST#8644S), Rabbit phospho-Rb (Ser807/811) (1:3000, #8516S), Mouse anti-Rb (4H1) (1:3000, CST# 9309S), Rabbit anti-cyclin D1(1:3000, Abcam#49604S), Rabbit anti-β-Actin (13E5) (1:5000, CST# 4970S). Secondary antibodies: Goat Anti-Mouse IgG H&L (HRP) (1:10000, Abcam#Ab6789), Goat Anti-Rabbit IgG H&L (HRP) (1:10000, Abcam#Ab205718).

### Gene expression analysis by RNA-sequencing

MCF7 were cultured in hormone deprivation media (with 10% charcoal-stripped FBS) for at least 3 days before assay. Trypsinized cells were seeded in a 12-well plate, stimulated with 1 nM β-estradiol (E2), and treated with/without 1 μM compounds for 24 h. Total RNA was extracted using the TRIzol reagent according to the manufacturer’s protocol. RNA purity and quantification were evaluated using the NanoDrop 2000 spectrophotometer (Thermo Scientific, USA). RNA integrity was assessed using the Agilent 2100 Bioanalyzer (Agilent Technologies, Santa Clara, CA, USA). Then the libraries were constructed using TruSeq Stranded mRNA LT Sample Prep Kit (Illumina, San Diego, CA, USA) according to the manufacturer’s instructions. The transcriptome sequencing and analysis were conducted by OE Biotech Co., Ltd. (Shanghai, China). The libraries were sequenced on an Illumina HiSeq X Ten platform and 150 bp paired-end reads were generated. About 48 ~ 50 M raw reads for each sample were generated. Raw data (raw reads) of FASTq format were firstly processed using Trimmomatic [26] and the low-quality reads were removed to obtain the clean reads. Then about 47 ~ 48 M clean reads for each sample were retained for subsequent analyses. The clean reads were mapped to the human genome (GRCh38) using HISAT2 [27]. FPKM [28] of each gene was calculated using Cufflinks [29], and the read counts of each gene were obtained by HTSeqcount [30]. Differential expression analysis was performed using the DESeq (2012) R package [6]. P value < 0.05 and foldchange > 2 or foldchange < 0.5 was set as the threshold for significantly differential expression.

### ER pathway activity evaluation

HCC1500, T47D, and EFM-19 were cultured in hormone deprivation media with 10% charcoal-stripped FBS for at least 3 days before assay. Trypsinized cells were seeded in a 12-well plate, stimulated with 1 nM E2, and treated with/without 1 μM compounds for 24 h. Total RNA was extracted using QIAGEN RNeasy Plus Mini Kit (Qiagen), according to the manufacturer’s protocol. The concentration of RNA samples was determined using NanoDrop 8000 (Thermo Scientific). SYBR Green expression assay (Qiagen#204154) was used to quantify GREB1 (Forward primer: CTGCCCCAGAATGGTTTTTA; reverse primer: GGACTGCAGAGTCCAGAAGC), AGR3 (Forward primer: GCTTTGGGTCTCTGCCTCTTAC; reverse primer: TTGACAATCCTCCAGGTGATGA) and the house-keeping genes actin (OriGene#HP204660). The relative quantities were determined using ΔΔ threshold cycle (ΔΔCt), according to the manufacturer’s instructions (Applied Biosystems).

### Cell cycle analysis

Trypsinized MCF-7 cells were seeded in a 24-well plate and treated with single or combined compounds for 40 h. Cells were collected and stained with 50 mg/mL propidium iodide (PI) solution in the presence of RNase (1 mg/mL) for 30 min on ice. The cells were then resuspended and analyzed with a FACS (BD Canto plus). At least 10,000 cells were counted for analysis.

### MCF-7 and T47D subcutaneous xenograft mouse models

Female Balb/c nude mice were used for MCF-7 and T47D tumor subcutaneous xenograft studies. At least one day before tumor cell implantation, estrogen pellets (0.18 mg, 17β-Estradiol, 60-day release, Innovative Research of America, Sarasota, FL, USA) were implanted subcutaneously. Each mouse was subcutaneously injected with 1 × 10^7^ MCF-7 (ECACC) or T47D cells in the right flank, and tumor growth was monitored. The long diameter (a) and the short diameter (b) of the tumor were measured using a caliper, and the tumor volume was calculated using the following formula: V = 0.5 × a × b^2^. When the average tumor volume reached 100–200 mm^3^ (designated as Day 0 of the study), the mice were randomly assigned to several groups of 8 animals each and treated with vehicle, Fulvestrant (250 mg/kg, subcutaneous injection, once a week), Palbociclib (40 mg/kg, oral gavage, daily), SCR-6852 (0.3, 1, 3, or 10 mg/kg, oral gavage, daily), or combinations as indicated in each figure. Tumor volumes were evaluated twice per week. Treatment tolerability was assessed by body weight measurements and frequent observation for clinical signs of treatment-related adverse effects.

### The intracranial MCF-7 tumor model

Female NPG mice were used for the intracranial MCF-7 tumor model. Three days before tumor cell implantation, estrogen pellets (0.72 mg, 17β-Estradiol, 60-day release, Innovative Research of America, Sarasota, FL, USA) were implanted subcutaneously. Each mouse was intracranially injected with 2 × 10^6^ MCF-7 (ATCC) cells. Eight days after tumor cell implantation (designated as Day 0 of the study), mice were randomized into four groups of 8 animals each and treated with vehicle, Fulvestrant (250 mg/kg, subcutaneous injection, once a week), or SCR-6852 (3, or 10 mg/kg, oral gavage, daily). Survival of mice was evaluated until Day 60. Mice were euthanized with weight loss exceeding 20% or moribund. On Day 60, all the mice remaining alive were euthanized. Before euthanasia, the mice were perfused with 4% paraformaldehyde and the brain tissues were collected for H&E staining using AUTOSTAINER XL (Leica, Wetzlar, Germany).

### Assessment of uterotropic activity

Female SD rats at 3 weeks of age with bodyweights ranging from 62.3 to 82.3 g were randomized into three groups of 8 animals each and treated with vehicle, tamoxifen (60 mg/kg), or SCR-6852 (10 mg/kg) by oral gavage once daily for three consecutive days. Twenty-four hours after the final dose, all animals were euthanized. Body weights and wet uterine weights were recorded for each animal. Fresh uterine tissue from each rat was fixed in 4% paraformaldehyde, dehydrated by HistoCore PEARL (Leica), and embedded by HistoCore Arcadia H + HistoCore Arcadia C (Leica). Sections were cut at 4 μm and stained with 0.1% toluidine blue O. The thickness of endometrial epithelium was measured using Leica Aperio CS2 with ImageScope × 64 program (Leica). The mean of five measurements per specimen was calculated.

### Brain distribution studies

MCF-7 tumor bearing mice were orally administrated with multiple doses of SCR-6852, AZD-9833 or GDC-9545 once a day at 10 mg/kg, respectively, or subcutaneously administrated with fulvestrant at 250 mg/kg once per week. The blood and brain tissues were collected at 24 h post the last dose. In addition, three male Sprague–Dawley (SD) rats (200–300 g, 6–8 weeks old, Beijing Vital River) were orally administrated with 10 mg/kg SCR-6852 and the blood and brain tissues were collected at 24 h post-dose. In a 14-day repeated oral dose-range finding study of SCR-6852 conducted in beagle dogs at 10 mg/kg/day, the blood and brain tissues were collected at 24 h post the last dose to determine the brain penetration of SCR-6852 in dogs. All blood was collected in K2-EDTA tubes and plasma was obtained by centrifuging the blood at 3200 rpm for 10 min at 4 °C and the brain was rinsed with water and blotted to remove superficial meninges. Plasma and brain samples were stored at − 80 °C until LC–MS/MS analysis which was performed on an Acquity UPLC system (Waters, USA) coupled to an AB SCIEX Triple Quad 6500 + System.

### Statistical analysis

Statistical and graphical presentations were performed using IDBS XLfit and GraphPad Prism 9. For cell proliferation assays, the IC_50_ was calculated by fitting a dose–response curve using a nonlinear regression model with a log(inhibitor) vs response curve fit. Relative IC_50_, determined as the concentration where 50% of the maximal response is observed, was calculated by the IDBS XLfit curve fitting software. Tumor growth inhibition (TGI) at the end of the study was calculated using the following formula: TGI (%) = (1-(*V*_*t* (treatment group) _− *V*_0(treatment group)_)/(*V*_*t*__(vehicle group) _− *V*_0(vehicle group)_) × 100%; *V*_0_ is the tumor volume of the animal when treatment starts; *V*_*t*_ is the tumor volume of the animal someday after treatment. The tumor volumes were analyzed by two-way ANOVA followed by Tukey’s multiple comparisons tests. The survival curves were analyzed by Log-rank (Mantel-Cox) test. The uterine relative weights and the thickness of endometrial epithelium were analyzed by one-way ANOVA followed by uncorrected Fisher's LSD. *P*-values < 0.05 were considered statistically significant.

## Results

### SCR-6852 demonstrates a fully antagonistic binding model to ERα

**Fig. 1:**
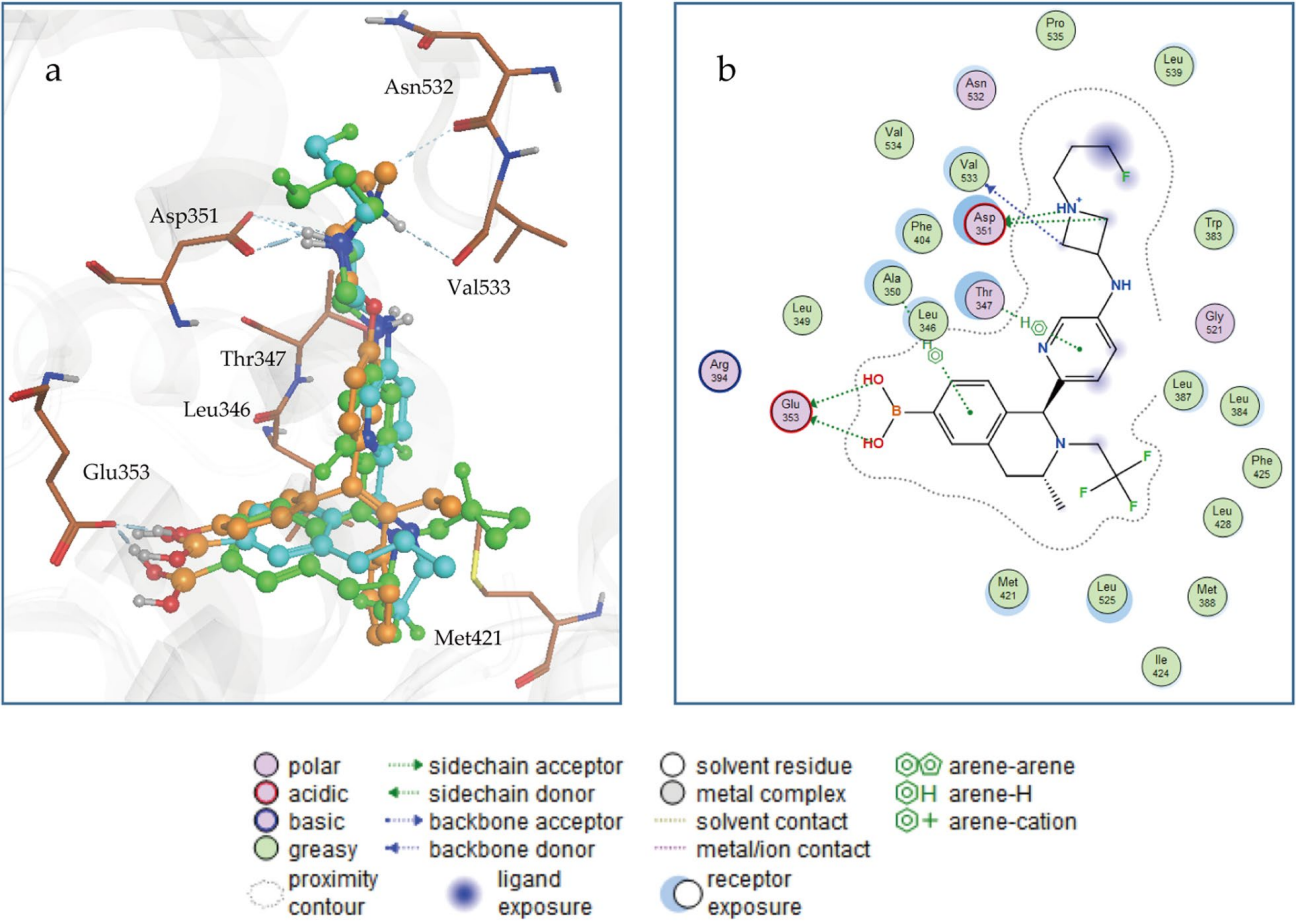
Docking diagram of SCR6139, SCR6515, and ERα-LBD complex. **a** SCR6139 and SCR6515 were docked to ERα-LBD complex (PDB: 6ZOQ; the crystal ligand in 6ZOQ (carmine), SCR6139 (blue), SCR6515 (green). **b** Schematic diagram of two-dimensional interaction for SCR6139

Based on the molecular binding mode of ERα with its ligand, a series of SERD molecules were rationally designed through the structure-based optimization strategy, such as SCR-6139, SCR-6515, and SCR-6852. According to the co-crystal structure of ERα and its ligand ((R)-2,3-dimethyl-2,3,4, 9-tetrahydro-1 h-pyrido [3,4-b] indole), this particular ligand demonstrated two key hydrogen bonds with Glu353 and Asp351, which were critical for antagonistic binding. Besides, additional hydrophobic interactions and Pi-Pi interactions among residues Phe40 and CH-Pi interactions with Thr347, Leu525 and Ile424 were also very important (Fig. 1a). Comparison with the well-defined binding mode of ERα- ligand, we identified that Fulvestrant, SCR6515, and SCR6139 were not only able to maintain the primary hydrogen bonding interactions with Glu353 residue but also increase the hydrogen bonding interactions with Asp351 and Val533 (Fig. 1, Additional file 1: Fig. S1). The docking scores are comparable at -9.59, -11.17, and -9.72 kcal/mol for Fulvestrant, SCR6515, and SCR6139, respectively (Table 1). This molecular docking indicated that both SCR-6515 and SCR-6139 are likely to have similar antagonistic effects to Fulvestrant. Additionally, SCR-6852, an analogue of SCR-6515 and SCR-6139 exhibited a similar binding model and equal level docking score of SCR6515 and SCR6139. The docking score for SCR-6852 is -10.38 kcal/mol (Table 1). Further, SCR-6852 also maintained the key interactions appeared in the Co-crystal, such as the hydrogen bonds with Asp351 and Val533 and Pi–Pi interaction with residue Phe404. These results suggested SCR-6852 probably could be a pure ERα antagonist. Furthermore, the antagonistic activities were identified on a nuclear translocation assay, SCR-6852 exhibited the pure antagonism activity with the maximal inhibition of 100% comparable to Fulvestrant (Additional file 1: Fig. S2). Meanwhile, the high selectivity (about 500-fold) of SCR-6852 against Progesterone Receptor (PR) or Androgen Receptor (AR) was obtained, and no obvious agonist/antagonist activities on several safety-relevant off-targets (Additional file 2: Table S1, SAFERYscan E/IC50 ELECT-78 assays, by Eurofins Discovery).

**Table 1:** The docking score for the six ERα antagonists

	6ZOQ-lig	4-hydroxy tamoxifen	SCR6139	SCR6515	Fulvestrant	SCR6852
DS^*^(kcal/mol)	− 8.85	− 6.83	− 9.72	− 11.17	− 9.59	− 10.38

*: Docking score used in MOE (GBVI/WSA dG)

### SCR-6852 effectively degrades ERα and inhibits the growth of ER + cell lines

**Fig. 2:**
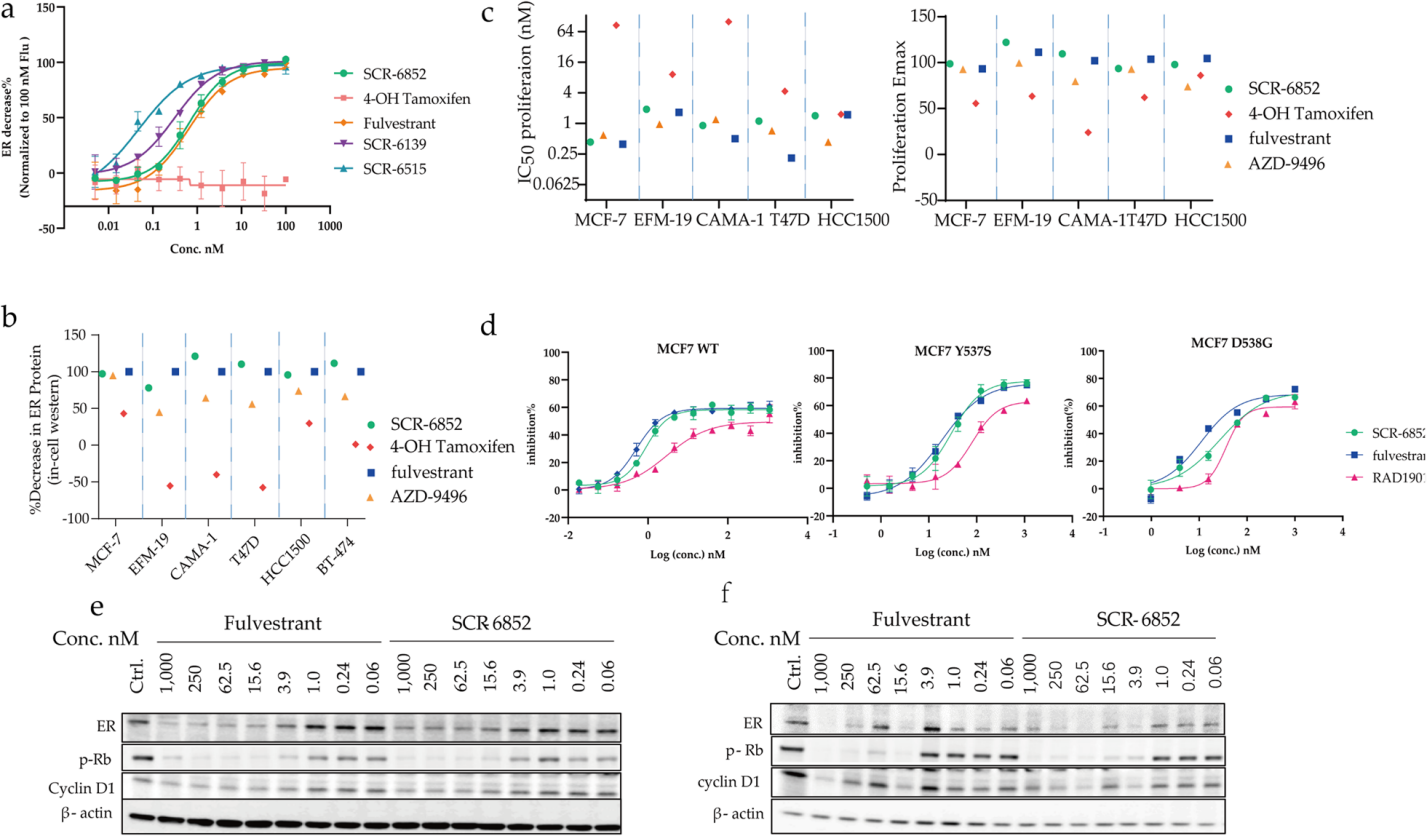
SCR-6852 is a potent SERD to induce ERα degradation and anti-proliferation of ER + breast cancer cell lines. **a** The comparison of in-house compounds potency to fulvestrant or 4-OH-tamoxifen by In-Cell Western Assay (ICW). MCF7 cells were seeded in a 384-well plate and linear-dilution compounds were administrated in duplicate for each treatment. After incubation for 24 h, Cells in the assay pate were treated as described in methods. ERα levels were quantified by immunofluorescence assay. 100% normalized to fulvestrant activity at 100 nM. Data are given as mean ± SEM. **b** The maximal ERα degradation across an ER-positive cell panel was evaluated in an In-cell Western assay. Cells were dispended into 96-well plates and incubated for 24 h with compounds (100 nM) treatment. Cellular ERα levels in each treated well were quantified by immunofluorescence assay.100% normalized to fulvestrant activity at 100 nM. **c** The comparison of cell viability between SCR-6852, AZD-9496, fulvestrant, or 4-OH-tamoxifen activity, respectively, across an ER-positive cell panel. Cells were seeded in 384-well plates and treated with linear-dilution compounds for 7 days of incubation. Cell viability was assessed using CellTiter-Glo. Cell growth inhibition is presented as a percentage of CellTiterGlo activity relative to the vehicle control. 100% normalized to maximal fulvestrant activity. **d** The comparison of cell viability between SCR-6852, fulvestrant or RAD-1901 activity, respectively, in MCF7 cells with ER WT, or mutant ESR1 Y537S, or mutant ESR1 D538G with the same operation as above. **e**–**f**, The evaluating of ERα level, as well as RB phosphorylation and cyclin D1 as ER targets by western blot, in MCF7 ER. WT (**e**) and ER. Y537S cells (**f**). Cells were seeded in 6-well plates and treated with linear-titration Fulvestrant or SCR-6852 for 5 days of incubation. Then cells in each well were collected and the targeting proteins in lysate supernatant were detected by WB

Cellular data demonstrated that all these compounds, SCR-6139, SCR-6515 and SCR-6852 effectively induce ERα degradation in MCF7 breast cancer cells (ERα wild-​type, wt), as shown in (Fig. 2a, Table 2, Additional file 1: Fig.S3). In particularly, SCR-6852 induced ERα degradation at the nanomolar concentrations (half maximal degradation concentration [DC_50_] of 1.05 ± 0.35 nmol/L), with the maximal ER degradation activity (Dmax) of 57.6%. In comparison of Fulvestrant, SCR-6852 demonstrated comparable ERα degradation capacity either at the DC_50_ or at the Dmax of degradation. The maximal ERα degradation activities of SCR-6852 were further evaluated in a panel of ER + breast cancer cell lines. As shown in Fig. 2b, SCR-6852 achieved a consistent maximal ERα degradation rate across those lines and showed comparable activities to Fulvestrant. In parallel, AZD9496, an incomplete ERα degrader showed a less potency ERα degradation rate. Consistent with ERα degradation, SCR-6852 strongly inhibited those ER + lines proliferation and achieved comparable activities with Fulvestrant either at IC_50_ or at the Emax (Fig. 2c). Although AZD9496 showed comparable activities at IC_50_, it achieved lower maximal anti-proliferation rate in two cell lines, CAMA-1 and HCC1500. In addition, 4-OH Tamoxifen (4-OHT), a Selective ER Modulator (SERM) showed less potent than all tested SERDs, suggesting ERα degrader rather than antagonist would achieve superior efficacy. Furthermore, SCR-6852 exhibited no inhibitory effect on the growth of ER- cell line (SK-BR-3) even at a high concentration (2 µM/L), suggesting the high selectivity of SCR-6852 for ERα-dependent tumor cells (Additional file 1: Fig. S4).

**Table 2:** In vitro properties of SCR-6852

	ERα degradation assay			Viability assay		
	DC_50_ nM	D_max_ [% Ful.]	D_max_ [% Veh.]	IC_50_ nM	E_max_ [% Ful.]	E_max_ [% Veh.]
MCF-7 WT						
Fulvestrant	0.93	97.69	56.04	0.40	97.88	49.60
SCR-6852	1.05	100.45	57.61	0.72	96.22	48.96
SCR-6515	0.08	97.83	66.81	0.29	103.00	64.15
SCR-6139	0.31	101.13	69.02	0.40	98.77	58.83
MCF-7 Y537S						
Fulvestrant	32.05	87.09	26.38	16.29	95.49	65.05
SCR-6852	47.32	99.57	30.17	27.6	100.96	68.78
MCF-8 D538G						
Fulvestrant				11.10	106.1	68.57
SCR-6852				26.96	109.5	70.8

ERα mutations with gain-of-function capabilities have been shown to be one of the resistance mechanisms to anti-ERα therapies in patients with breast cancer [4]. SCR-6852 strongly inhibited the proliferation of ERα wt (MCF7 parental) and ESR1 mutants Y537S/ D538G (MCF7 ESR1 Y537S or MCF7 ESR1 D538G) cell lines, which was comparable with Fulvestrant (Fig. 2d). Meanwhile, comparison with RAD1901, an approved oral SERD recently, SCR-6852 was more potent on inhibition of cell growth both in ESR1 WT and mutant lines (Fig. 2d). Furthermore, the degradation activities of SCR-6852 on ERα in ESR1 Y537S mutant and the effects of the downstream signals were determined by western blot. SCR-6852 dose-dependently degraded both WT (Fig. 2e) and Y537S mutant (Fig. 2f) ERα in cells, which showed comparable potency with Fulvestrant. The key regulators of ER signal axis, cyclin D and phosphorylated retinoblastoma (pRb) were both downregulated following the ERα degradation (Fig. 2e, f and Additional file 3).

**Fig. 3:**
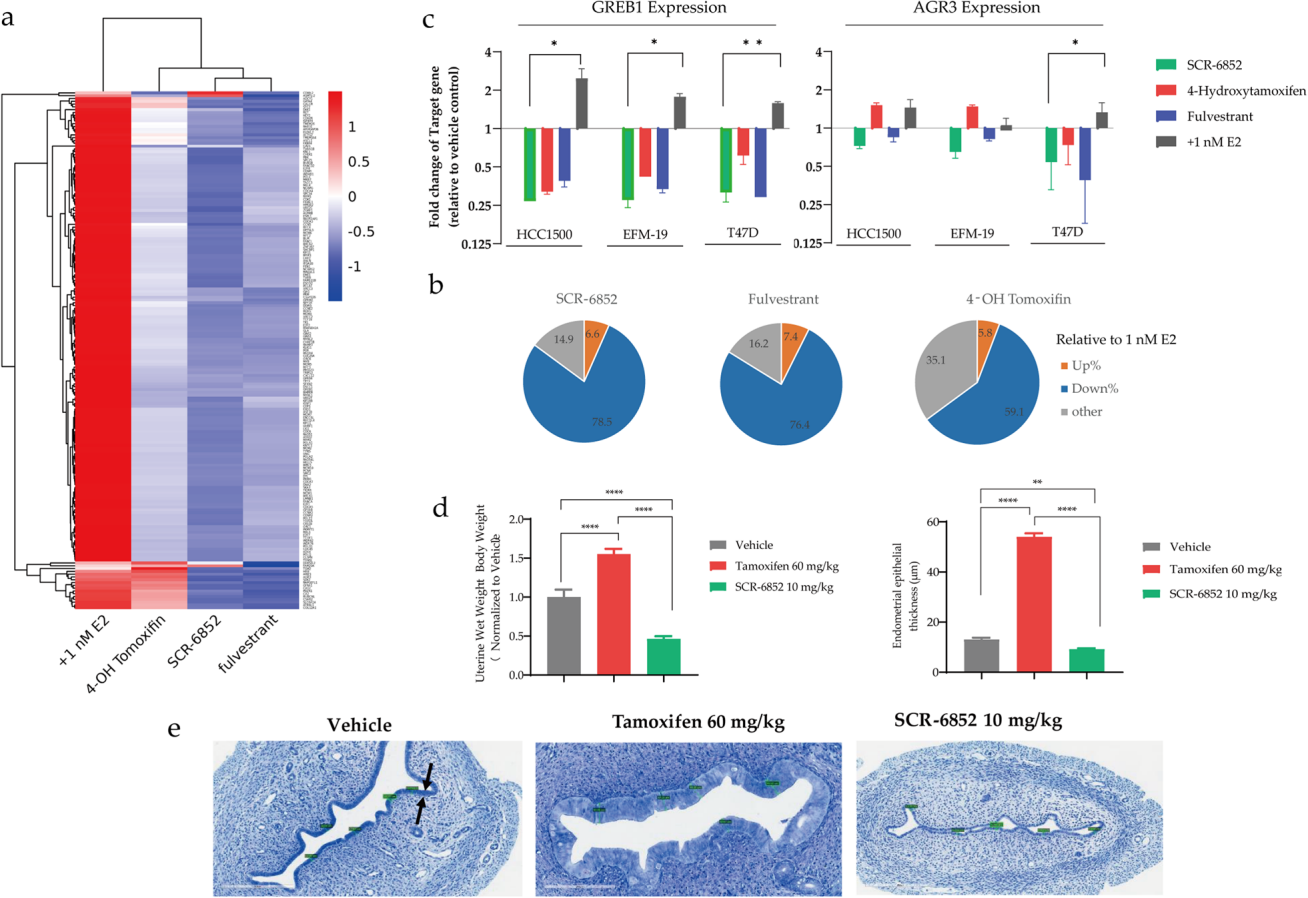
Effects on ER target genes expression and uterine tissue of SCR-6852. **a** Differential expression analysis from RNA-seq. MCF7 with hormone-deprived pre-treatment were administrated ER ligands or not present with 1 nM E2 stimulation for 24 h incubation in a 12-well plate. Total mRNA was isolated from treated cells and sequenced on an Illumina HiSeq X Ten platform. FPKM of each gene was calculated using Cufflinks and Differential expression genes analysis by using the DESeq (2012) R package. Four separate clusters were obtained. Rows show genes up- or downregulated by E2 relative to control (> twofold, FDR < 0.05), or by each ER ligand. **b** Summary of the transcriptional consequences. In orange is the percentage of upregulated genes compared with E2 in total test genes by experimental ligand (> twofold, FDR < 0.05), and those in blue are downregulated. **c** ER-target gene *GREB1* or *AGR3* expression analysis. T47D EFM-19 and HCC1500 cells were grown in RPMI media supplemented with 10% charcoal-stripped FBS for 14 days and then treated with ER antagonist (1 μM) or DMSO in the presence E2 (1nM) stimulation for 24 h before RNA isolation and gene expression analysis as described in methods. Asterisks show one-tail *t*-test *p*-values. *P*-values: * *P* < 0.05; ** *P* < 0.01. **d-f** Effect of SCR-6852 on uterine tissue. 21 days old SD rats were dosed with a vehicle, 60 mg/kg tamoxifen or 10 mg/kg SCR-6852 orally every day for three days. The uteruses were harvested 24 h after the final doses and stained by toluidine blue. Endometrial height was assessed from the basement membrane to the luminal border (scale bar in green lines: 200 μm), Arrows indicate the uterine epithelium (**e**). Wet uterine weight normalized to body weight and Endometrial thickness were digitally measured and plotted in the graph in figure (**d** and **e**). * *P* < 0.05; ** *P* < 0.01; *** *P* < 0.001; **** *P* < 0.0001

The estrogen receptor is a ligand-inducible transcription factor that regulates the transcription of numerous genes. To explore the impact of SCR-6852 on the ERα-target genes transcription, transcriptomic analysis was performed in MCF7 cells in the presence of E2, SCR-6852, Fulvestrant, and 4-OHT, respectively. MCF7 were hormone-deprived before ER ligand treatment and then differentiated gene transcription was compared to that of E2-stimulation. Results from Principal component analysis (PCA) showed that the gene transcriptomic data in cells treated with SCR-6852 and Fulvestrant, respectively, were clustered and differed from the 4-OHT treatment group. It was observed that 4-OHT partially promoted transcription of some ER target genes. Results from transcriptomics showed that 5.8% of the genes induced by E2 also being upregulated by 4-OHT (foldchange > twofold), and 59.1% of E2-induced genes were suppressed by 4-OHT (Fig. 3a, b, Additional file 2: Table S2). In contrast, SCR-6852 and Fulvestrant inhibited the transcription of 78.5% and 76.4% of E2-induced genes, respectively. Furthermore, two typical ER target genes, *AGR3* and *GREB1* were chosen to test the effect of SCR-6852 on the E2-induced gene transcription by RT-QPCR in more ER + cell lines. Consistent with transcriptomic data from MCF7, both Fulvestrant and SCR-6852 significantly downregulated both *AGR3* and *GREB1* transcription in T47D, EFM-19, and HCC1500 cells (Fig. 3c). while a significant increase of *AGR3* transcription was observed in both EFM-19 and HCC1500 by 4-OH-tamoxifen treatment. Taken together, SCR-6852 is a pure ERα antagonist.

Since the activity of ER ligands could be tissue-dependent, we next evaluated the effects of SCR-6852 on the uterus of juveniles in rats. As shown in Fig. 3d, Tamoxifen at 60 mg/kg resulted in a significant increase in relative uterine wet weight (*P* < 0.0001). In contrast, oral administration of SCR-6852 at 10 mg/kg decreased the uterine wet weight instead (*P* < 0.0001). Treatment-dependent changes in the uterine tissue were further investigated by quantitative microscopic histological analysis. As shown in Fig. 3e and f, SCR-6852 at 10 mg/kg decreased the endometrial epithelial thickness compared to vehicle treatment (*P* < 0.01), while tamoxifen at 60 mg/kg significantly increased the endometrial epithelial thickness (*P* < 0.0001). These data suggested that SCR-6852 completely antagonized the ER signal axis in the uterine tissue.

### SCR-6852 exhibits superior anti-tumor activities in the ER + subcutaneous xenograft breast cancer tumors

**Fig. 4:**
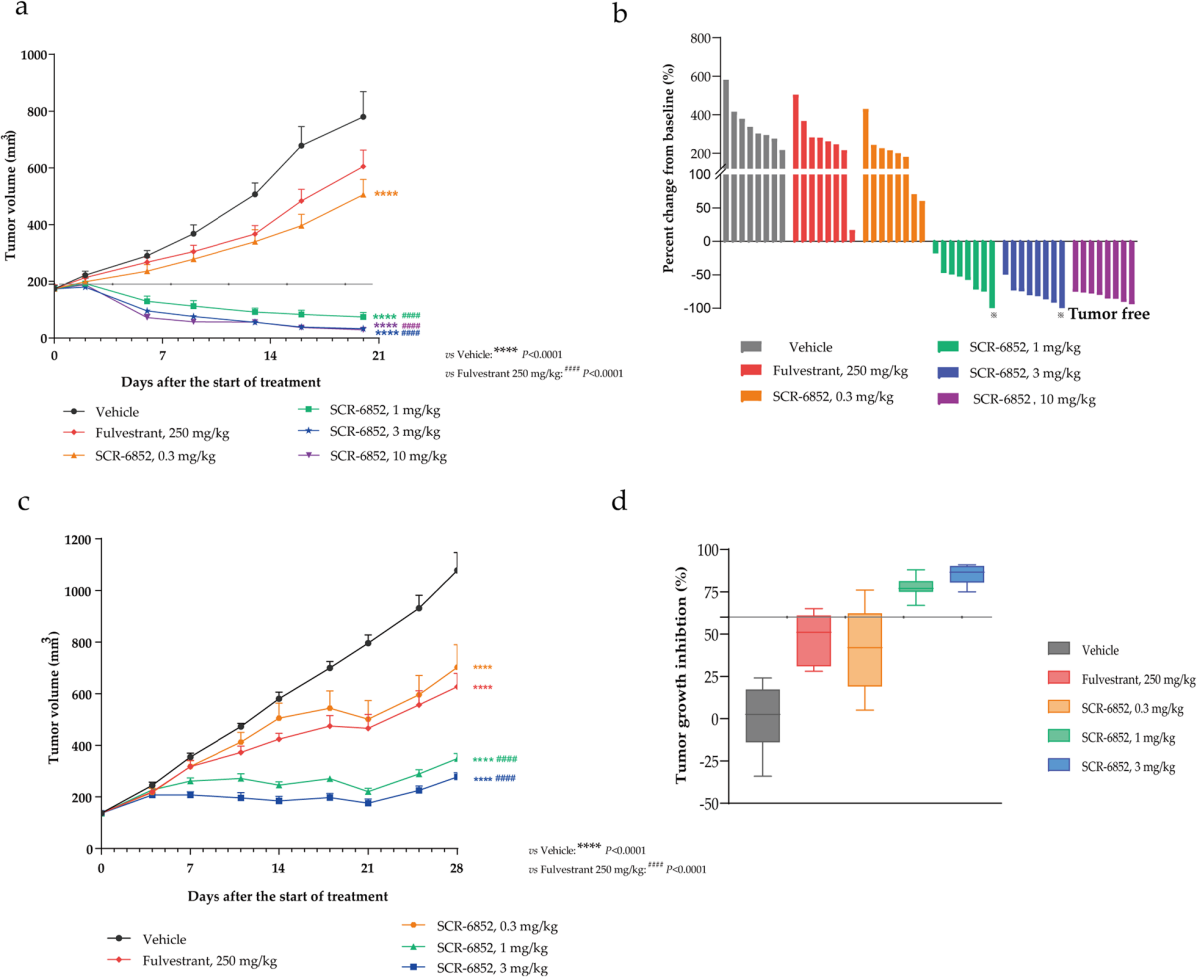
Antitumor activity of SCR-6852 in xenograft models. **a**–**b** ER-positive cancer cell line MCF-7 was implanted in Balb/c nude mice with 17β-Estradiol to stimulate tumor growth. Animals were treated with SCR-6852 (0.3, 1, 3, or 10 mg/kg, respectively, daily, orally) or 250 mg/kg fulvestrant (subcutaneous injection once a week). Tumor volume was evaluated twice per week until the study endpoint. **a** Mean tumor volume ± SEM. **b** Percent change in tumor volumes from individual animals from the start of treatment to the end of treatment. **c**–**d** T47D subcutaneous xenograft model was also used. Tumor-bearing Balb/c nude mice received the vehicle, 250 mg/kg fulvestrant, or SCR-6852 0.3, 1, 3 mg/kg (QD) (n = 8/group). **d** Tumor growth inhibition for each treatment group relative to vehicle at end of treatment. The error bars represent the standard error of the mean (SEM). **** *P* < 0.0001 versus vehicle, ^####^ *P* < 0.0001 versus Fulvestrant

The in vivo antitumor activities of SCR-6852 were evaluated in two ER + breast cancer subcutaneous tumor models. SCR-6852 dose-dependently inhibited MCF7 tumor growth (Fig. 4a), with TGIs being 45.22%, 116.33%, 123.26%, and 123.80% at 0.3, 1, 3, and 10 mg/kg, respectively (*P* < 0. 0001 for all SCR-6852 treatment groups compared to vehicle group). Meanwhile, SCR-6852 at the dosages of 1, 3, and 10 mg/kg all showed superior anti-tumor activities than Fulvestrant at 250 mg/kg (TGI of 28.84%, *P* < 0.0001), and one out eight mice with tumor-free were observed in 1 mg/kg and 3 mg/kg SCR-6852 treatment groups, respectively (Fig. 4b). Treatment of SCR-6852 was well tolerated with no significant bodyweight loss in animals (Additional file 1: Fig. S5a). Furthermore, to fully understand the effects of SCR-6852 on ER signal axis in tumors, a separate experiment was carried out for PD marker detection. The ER-target gene *PGR* [31] was significantly reduced in SCR-6852 treatment groups (> 1.8 mg/kg groups, *P* < 0.001) (Additional file 1: Fig. S6).

Additionally, in another ER + breast cancer cell T47D subcutaneous xenograft tumor model, SCR-6852 demonstrated robust tumor growth inhibition with TGI of 77.47%, and 85.04% at 1 and 3 mg/kg, respectively, presenting superior anti-tumor activities than fulvestrant at 250 mg/kg (TGI of 48.35%, *P* < 0.0001, Fig. 4c and d). Even at a very low dose, 0.3 mg/kg, SCR-6852 demonstrated comparable efficacy with Fulvestrant at 250 mg/kg (TGI: 39.87% Vs 48.35%). Again, SCR-6852 was tolerated well, and there was no significant body weight loss observed in all test animals (Additional file 1: Fig. S5b).

### SCR-6852 has high brain penetrability and effectively suppresses ER + tumor growth in an intracranial tumor model.

**Fig. 5:**
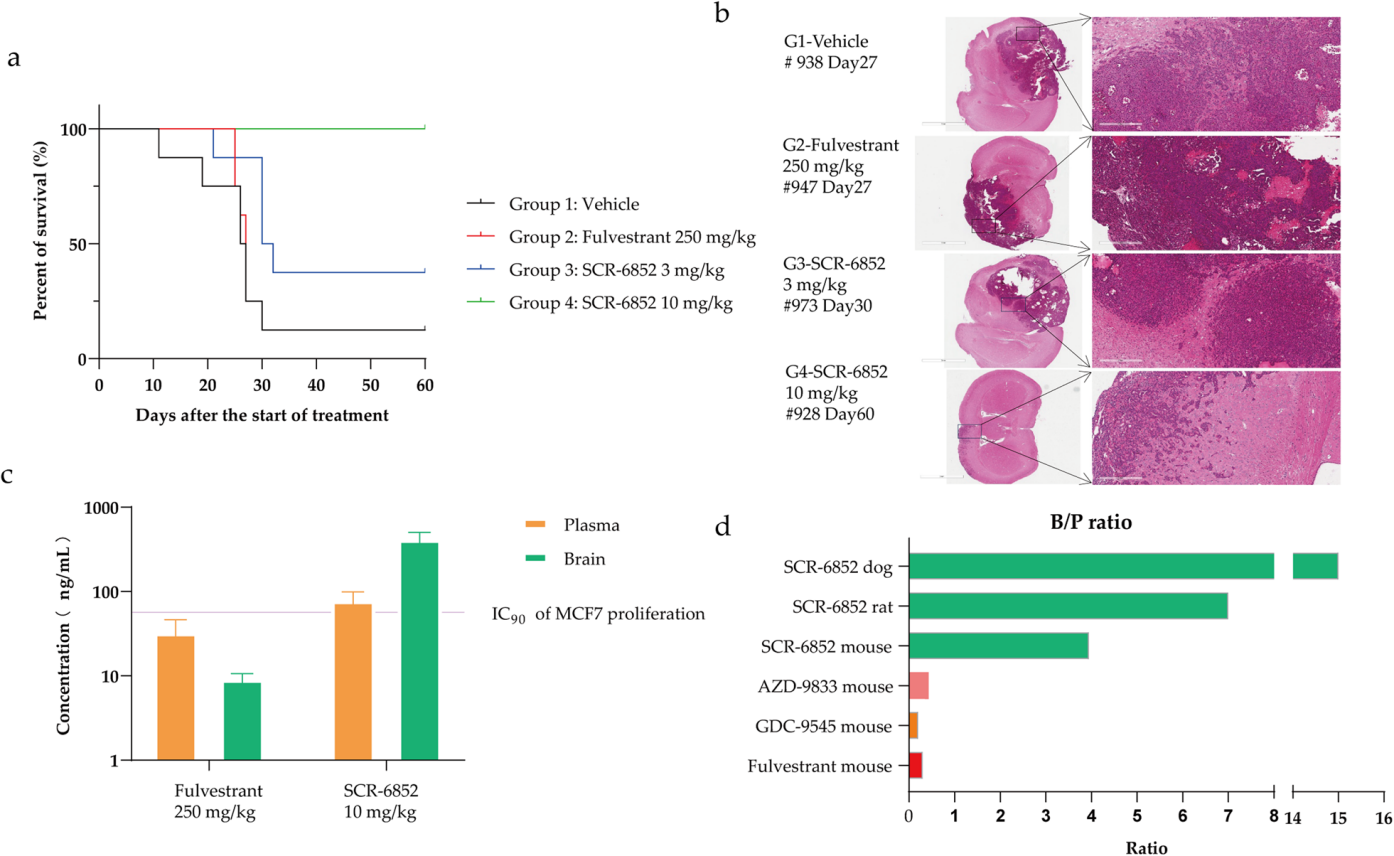
BBB permeability and in vivo activity. **a**–**b** Female NPG mice were implanted with MCF-7 cells intracranially. Eight days after tumor cell implantation (designated as Day 0 of the study), mice were treated with vehicle, 250 mg/kg fulvestrant (administration as described above), 3 or 10 mg/kg SCR-6852 (QD, *n* = 8/group), and survival of the animals were recorded. The survival curves of the animals are shown (**a**). Representative images of the brain tissue H&E staining are also shown (**b**). The day that the brain tissues were collected for the representative images is indicated in the graph. Scale bar: 3 mm or 300 μm. **c** The intracranial MCF7 (orthotopic) xenograft mice were treated with SCR-6852 or Fulvestrant for 8 days, and the tissues were collected 24 h after the last dose. SCR-6852 or Fulvestrant concertation in the brain or plasma was determined by LC–MS. Brain conc./plasma conc. was presented as a B/P ratio. **d** The CD1 IGS mice/rat / Beagle were administrated with multiple doses of SCR-6852 or other compounds, and the tissues were collected at 24 h after the last dose as described in methods. relative compound concertation in the brain or plasma was determined by LC–MS. Brain conc./plasma conc. was presented as a B/P ratio

The inhibitory activity of SCR-6852 on the tumor metastasis in the brain was evaluated in an intracranially orthotopic xenograft model. The ER + MCF7 cells were intracranially implanted and anti-tumor efficacy was evaluated using survival as the primary endpoint based on the Kaplan–Meier survival analysis. As shown in Fig. 5a and Additional file 1: Fig.S5c, SCR-6852 dose-dependently increased the mice survival, and the median survival time of mice received 10 mg/kg of SCR-6852 treatment significantly increased (not reached) compared to the vehicle group (26.5 days) (*P* < 0.001). Notably, all mice that received 10 mg/kg of SCR-6852 treatment survived by the end of the study (Day 60). In parallel, mice received 250 mg/kg of Fulvestrant treatment had no significant difference in median survival time compared to vehicle (27 vs. 26.5 days, *P* > 0.05), although it achieved moderate anti-tumor activity in subcutaneous tumors. Next, infiltration and proliferation of tumor cells in these mice's brain tissues were evaluated by H&E staining. The brain tissues were harvested when mice are sacrificed due to tumor progression or at the end of the study if the mice remained healthy (Day 60). As shown in Fig. 5b, significant tumor cells infiltration and destruction of physiological structure were observed in vehicle, Fulvestrant, and 3 mg/kg of SCR-6852 treatment groups, while only a very small amount of tumor cell infiltration was observed in the brain tissues from 10 mg/kg of SCR-6852 group, confirming the robust anti-tumor efficacy of SCR-6852 in mice’s brain tissues.

Next, the exposure of SCR-6852 and Fulvestrant in brains were determined in the above intracranial MCF7 (orthotopic) xenograft model in an independent assay. Mice were administrated with SCR-6852 (10 mg/kg, oral garage, daily, from Day 0 to Day 7) or Fulvestrant (250 mg/kg, subcutaneous injection, once a week, on Day 0 and Day 7) (Fig. 5c), respectively, then the plasma and brain tissues were collected at 24-h post-last dose. The drug concentrations were determined by LC–MS/MS. The concentration of SCR-6852 in plasma was 78 ± 21 ng/ml, and in brain was 419 ± 82 ng/ml. SCR-6852 exhibited high brain exposure with a B/P (brain/plasma) ratio of more than 5 folds, while Fulvestrant tended to be distributed in plasma with a low B/P ratio of fewer than 0.5 folds. In addition, SCR-6852 concentration in brain was much higher than the IC_90_ value of anti-proliferation determined in MCF7 cells (56.68 ng/mL, corrected by 98.8% PPB), suggesting the robust anti-tumor activity in intracranial tumors correlates well to the exposure of drug in brains.

In addition to mice, the brain exposure of SCR-6852 in rats and dogs was further determined. Animals received an oral administration of SCR-6852 at 10 mg/kg, then plasma and brain tissues were collected at 24-hour after 14 days of dosing. As shown in Fig. 5d, SCR-6852 demonstrated extremely high exposure in both species with a B/P ratio of 7 folds in rats and 15 folds in dogs, respectively. In parallel, the brain exposure of some ER degraders, including Fulvestrant and oral SERDs, AZD-9833, and GDC-9545 were compared with SCR-6852 in mice side by side. Results showed that neither Fulvestrant nor those oral SERDs could effectively distribute to brains with the B/P ratio less than onefold. Taken together, SCR-6852 demonstrated high brain exposure in three preclinical species, including rodents and non-rodents.

### SCR-6852 synergistically inhibits ER + tumor growth in combination with a CDK4/6 inhibitor in vitro and in vivo

**Fig. 6:**
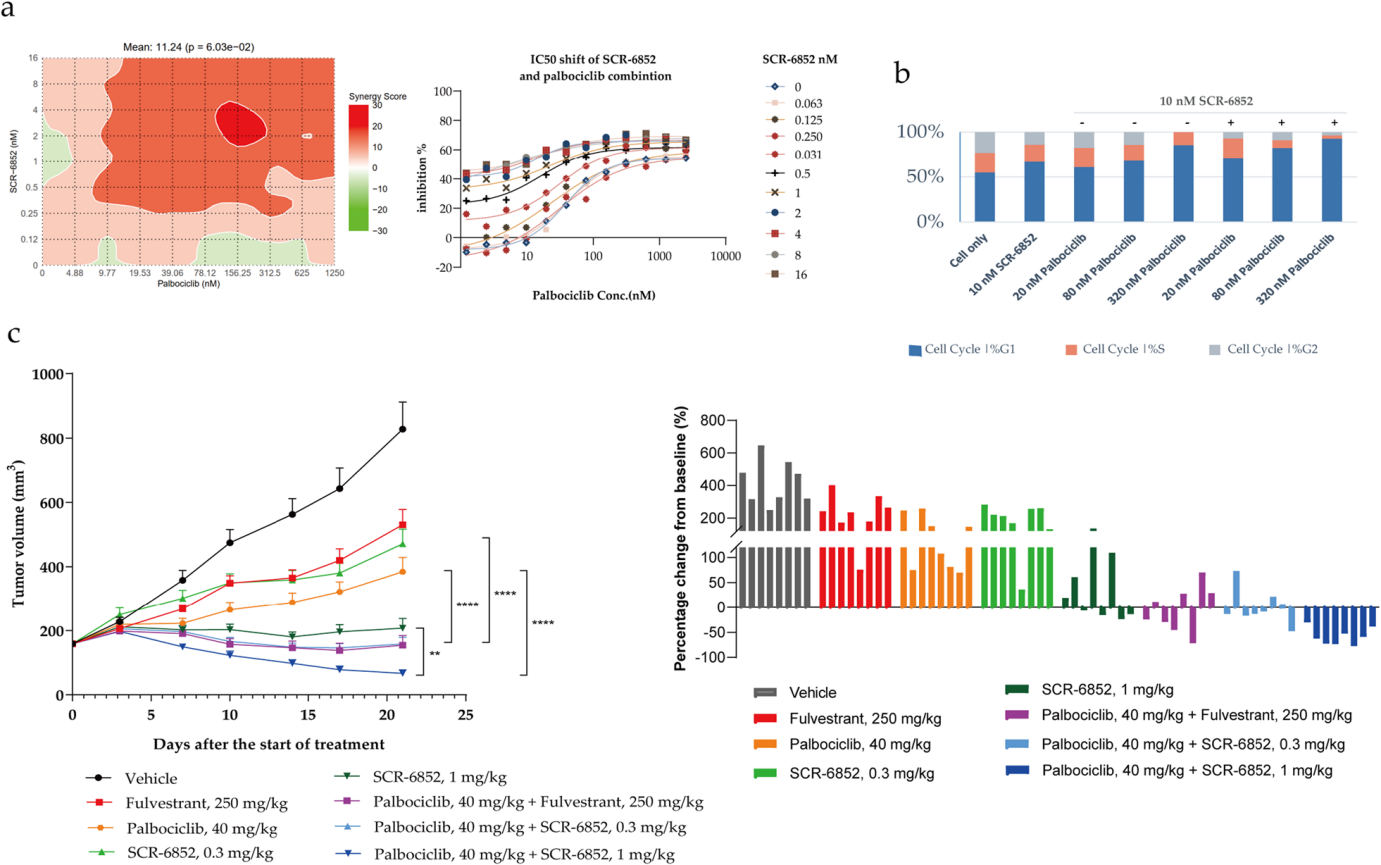
The synergistic effects for SCR-6852 combined with CDK4/CDK6 inhibitor in vitro and in vivo **a** MCF7 were treated with increasing concentrations of SCR-6852 and/or Palbociclib for 7 days in a 384-well plate. Cell viability was measured using Cell TiterGlo assay. Combination analysis with Loewe’s additivity mode by SynergyFinder (https://synergyfinder.fimm.fi) displayed surfaces of synergy on the left; red indicates synergy (synergy score > 0) and green indicates antagonism (synergy score < 0). The potency shift of Palbociclib combined with a serial SCR-6852 dosing were represented graphically as dose–response curves, on the right. **b** MCF-7 cells were treated with SCR-6852 or combined Palbociclib for 40 h, and cell cycle distribution was analyzed by Flow cytometry. The result of one representative assay from three similar independent experiments is shown. The percentages of cells in G1, S, and G2/M were shown as indicated. **c** The MCF-7 tumor-bearing Balb/c nude mice received the vehicle, 250 mg/kg Fulvestrant, 40 mg/kg Palbociclib, 0.3 mg/kg SCR-6852, 1 mg/kg SCR-6852, or combination treatments as indicated in the graph (*n* = 8/group). The error bars represent the standard error of the mean (SEM). ****P < 0.0001; **P < 0.01 combination versus single as indicated

Combination of the CDK4/6 inhibitor (CDK4/6i) and endocrine therapy (ET) has become standard treatments following the progression of initial AI monotherapy. Here we observed the synergistically anti-tumor effects of SCR-6852 in combination with a CDK4/6 inhibitor, Palbociclib. The synergy analysis displayed that the synergism in preventing the cells proliferation as SCR-6852 combined with Palbociclib was demonstrated with an average synergy score of 11 (Fig. 6a). And the combination with SCR-6852 significantly improved the anti-proliferation activity of Palbociclib with the apparent tenfold shift of IC_50_. Further cell cycle analysis showed that the combination of SCR-6852 and Palbociclib significantly increased the cell numbers in the G1 phase compared to SCR-6852 or Palbociclib alone. And accompanying the decrease of cell numbers in the S or G2/M phase was also observed (Fig. 6b).

The combination of SCR-6852 and Palbociclib was further assessed in the MCF-7 subcutaneous tumors. As shown in Fig. 6C, the combination of SCR-6852 (0.3 mg/kg) and Palbociclib (40 mg/kg) significantly enhanced the tumor growth inhibition compared to monotherapy (*P* < 0.0001, TGI: 100.10% vs. 53.35% vs. 66.34%; Groups: combo vs. SCR-6852 vs. Palbociclib). Similarly, the combination of 1 mg/kg SCR-6852 and 40 mg/kg Palbociclib also showed improvement in tumor growth inhibition compared to monotherapy (*P* < 0.05, TGI:113.76% vs. 92.70% vs. 66.34%; combo vs. SCR-6852Vs Palbociclib). Also, all treatments were well tolerated (Additional file 1: Fig. S5d).

## Discussion

The ER-target therapy that directly opposes the mitogenic action of estrogen or that block estrogen synthesis is an approved strategy for the treatment of ER-positive breast cancer. Here we identified a series of novel ER degraders such as SCR-6139 and SCR-6852. The modeling study of these compounds binding to ERα revealed that the molecular interactions of our compounds with the ERα ligand binding domain were nearly identical to those of Fulvestrant, indicating that these compounds were ERα degrader with potential pure-ER antagonistic activity. The following cell-based efficacy studies well demonstrated that one of those, SCR-6852, was a potent SERD that induced ER degradation and inhibited ER + cancer cell growth with a high potency comparable with Fulvestrant. Furthermore, in a parallel comparison assay, SCR-6852 displayed greater capabilities in ERα degradation and anti-proliferation than 4-OHT and AZD-9496 in multiple ER + breast cancer cell lines, indicating SCR-6852 was different from 4-OHT or AZD-9496 and similar with Fulvestrant. And the following transcriptional signatures also revealed that SCR-6852 cluster close to Fulvestrant, while tamoxifen regulated a subset of genes in a similar manner to estradiol. Particularly, the agonist activity on *AGR3* expression of tamoxifen was observed, and on the contrary, SCR-6852 downregulated this gene expression to the maximum level. Tamoxifen, as a SERM, had been well demonstrated robust antagonist activity in the breast epithelium but mimicked the agonist effect of estrogen in bone, endometrium, and serum lipid profiles [32–34]. Our study also verified that tamoxifen acted as a pro-estrogen agonist by causing the endometrium to appear thickened in immature rats, however, SCR-6852 decreased the uterine wet weight and endometrium thickness instead. This antiestrogenic effect of SCR-6852 was observed in Fulvestrant treatment in a previous report [35]. The above data demonstrated our compound SCR-6852 is a pure ER antagonist.

The acquisition of ligand-independent *ESR1* mutations during aromatase inhibitor therapy in metastatic ER + breast cancer was a common mechanism of hormonal therapy resistance [36]. The most common mutations in *ESR1* occurred at the Y537 and D538 residues, and the Y537S mutation was relatively more resistant to growth inhibition when treated with ER antagonists compared with D538G and WT [37]. In the MCF7 cells with *ESR1* Y537S mutation, SCR-6852 efficiently induced the mutant ERα degradation and demonstrated comparable anti-proliferation activities with Fulvestrant. Meanwhile, compared with RAD1901, SCR-6852 displayed more activity of anti-proliferation of both *ESR1* WT and Y537S/D538G mutant stain. Elacestrant was currently approved, and the efficacy and safety were evaluated in EMERALD (NCT03778931). In this study patients including WT or mutant *ESR1*, progressed on up to 2 lines of ET with a CDK4/6 inhibitor, and were randomized to either Elacestrant or standard-of-care ET (Fulvestrant, Anastrozole, Letrozole, or Exemestane). The positive clinical results were disclosed in ASCO, 2022. EMERALD met both of its pre-specified primary endpoints of progression-free survival (PFS) in the overall population and in patients with the *ESR1* mutation (*mESR1*) compared to SOC endocrine monotherapy. The PFS rate at 12 months with Elacestrant was 22.32% vs. 9.42% with SOC in the overall population and 26.76% vs. 8.19% in the ESR1 mutation population. These clinical trial data showed that Elacestrant reduced the risk of disease progression or death by 30% in all patients and by 45% in patients with ESR1 mutation. This significant efficacy in the Elacestrant arm demonstrated that an oral SERD was useful and better than a Fulvestrant for patients with *ESR1* mutants.

The treatment of breast cancer brain metastases is especially challenging. The blood–brain barrier restricts the diffusion of many drugs into the brain inclusive of limiting highly permeable drugs by active efflux transporters expressed in BBB. Therefore, effective exposure to drugs in the brain is critical to treat brain metastasis. Abemaciclib [38] significantly increased survival in a rat orthotopic U87MG xenograft model by confirming a sufficient unbound brain concentration with a target engagement ratio (the ratio of the unbound brain concentration to the in vitro enzyme IC_50_) of 3.3–14.3 for approximately 12 h in mice dosed with 30 mg/kg. And Buparlisib ([39]) is demonstrated as a brain penetrable panPI3K inhibitor for high brain accumulation (B/P ratio > 1.5) and efficient intracranial target inhibition at clinically achievable. These two clinical agents are now being investigated in clinical trials for BCBM treatment ((NCT02675231, NCT02437318) [40]. In this study, we revealed that SCR-6852 highly accumulated in the brain in mice. The brain concentration was more than sevenfold IC_90_ value of MCF7 growth inhibition. This sufficient SCR-6852 in brain leads to efficiently shrink tumor cells growth and a significantly prolonged mice survival (100% animals survival treated with 10 mg/kg by the end of the study) in MCF-7 intracranial tumor model. Additionally, we observed that SCR-6852 not only accumulated in mice brain but also in rats, in dogs with a B/P ratio of 4, 7, and 15, respectively. SCR-6862 demonstrated consistent and high brain penetrability across multiple pre-clinical animal species, supporting it could penetrate well into human brain. Although the pre-clinical study [41] reported that the detectable Elacestrant in the intracranial tumor (the B/P ratio was about 0.6 according to reported data) which was most possibly less than anti-proliferation IC_50_ value, and the further efficacy study demonstrated only 43% animals survived to the end of the study at day 54. And up to now, there was no case with brain metastasis reported benefit from Elacestrant treatment in the clinic. The brain metastasis incidence of ER + /HER- breast cancer patients was 5 ~ 10% as reported in a previous study [42]. Since clinical studies had found that there existed some discordance for receptor phenotype between the primary tumor and systemic metastases. Kaidar-Person et al. [43] performed multi-institutional data analysis and revealed that the discordance rates for the expression of ER between the primary breast tumor and subsequent BM were 12% (for 20 patients in 167). In particular, 44 in 57 primary ER + patients (77%) maintained the ER expression in BCBM. Overall, ER-target therapy was still needed and meaningful for BCBM patients. SCR-6852 had higher brain exposures and consistent BBB penetration capability in preclinical species, supporting a high potential for clinical application in BCBM with ER-positive expression.

Endocrine therapy combined with CDK4/6 inhibitors is now the standard first-line treatment option for patients with HR + /HER2– mBC and may also be considered a standard option in the second-line setting, given the significant improvements in PFS and OS [44]. A phase III study investigated Fulvestrant plus Palbociclib in advanced HR + /HER2- breast cancer that had progressed on prior ET or recurred within 12 months of stopping adjuvant ET. Despite being heavily pretreated with ET, adding Palbociclib to Fulvestrant still more than doubled patients' PFS (11.2 vs. 4.6 months; HR = 0.50 [95% CI = 0.40–0.62], *P* < 0.0001) and had prolonged OS by 10 months (median 39.7 vs. 29.7 months; HR = 0.72 [95% CI = 0.55–0.94]) [45]. In this study, the synergistic effects of SCR-6852 and Palbociclib were well demonstrated in anti-MCF7 growth in vitro and tumor inhibition in the MCF7 xenograft model. These results would support a combination therapy of SCR-6852 and CDK4/6 inhibitor in the clinical trial. Additionally, research revealed that tumor cells resistant to CDK4/6i could continue to rely on the ER pathway to drive tumor growth [46]. A clinical retrospective analysis demonstrated that hormonal therapy was effective, leading to significant PFS, in patients after Palbociclib progression [47]. The clinical benefits would be promising for SCR-6852 in CDK4/6 sensitive and resistant patients.

## Conclusion

Based on structural optimization, we identified a novel SERD, SCR-6852. High potency and efficacy on ER degradation and cell growth inhibition of SCR-6852 were verified in multiple ER-positive cell lines with ESR wt or mutant. As an ER degrader, SCR-6852 exhibited a pure ER antagonistic efficacy on ER target genes expression and had no agonistic effects on endometrium that was different from SERM. In the xenograft model, oral administration of SCR-6852 demonstrated a relatively strong tumor growth inhibition with maximal TGI 123% (MCF7, 3, 10 mg/kg dosing). A combination of SCR-6852 and CDK4/CDK6 inhibitors revealed synergistically anti-tumor activities both in vitro and in vivo. Notably, SCR-6852 showed excellent brain penetration features of > 4 of a B/P ratio in multiple animal models. In an intracranial tumor model study, SCR-6852 concentrations in the brain were monitored at 419 ng/g (at 24 h for 7 days, 10 mg/kg dosing), which is much higher than the anti-proliferation IC_90_ value, and significantly prolonged survival was finally observed with minimal tumor cell infiltration in the brain.

In summary, SCR-6852 is an oral SERD with high potency and efficacy on ER degradation and ER-positive breast cancer cells growth inhibition and excellent brain penetration of more than 4 B/P ratio in pre-clinical animal models making it an attractive candidate for intracranially-targeted therapeutic strategies involving advanced breast cancer patients even with brain metastases.

### Supplementary Information

**Additional file 1:** Supporting docking figures and pharmacological data in vitro and in vivo.

**Additional file 2:** Target selectivity and transcription raw data.

**Additional file 3:** Raw data of WB gels.

## Data Availability

The datasets used and/or analyzed during the current study are available from the corresponding author on reasonable request.

## Abbreviations

ER: Estrogen receptor
ER +: Estrogen receptor positive
SERD: Selective estrogen receptor degrader or downregulator
ABC: Advanced breast cancer
BCBMs: Breast cancer brain metastases
ET: Endocrine therapy
AI: Aromatase inhibitors
SERM: Selective Estrogen Receptor Modulator
IC_50_/IC_90_: The concentration of a drug or inhibitor needed to inhibit a biological process or response by 50% or 90%
DC_50_: The half-maximal degradation concentration that makes a protein for 50% degradation
Emax: The maximal effect at high drug concentrations when all the receptors are occupied by the drug
GREB1: Growth regulating estrogen receptor binding 1
AGR3: Anterior gradient 3, protein disulphide isomerase family member
ESR1: Estrogen receptor 1
CDK: Cyclin-dependent kinase
B/P: Brain-plasma ratio
PFS: Progression-free survival
OS: Overall survival
SOC: Standard of care
