# Supplementary material for: SCR-6852, an oral and highly brain-penetrating estrogen receptor degrader (SERD), effectively shrinks tumors both in intracranial and subcutaneous ER + breast cancer models
Source: Breast Cancer Res. 2023 Aug 14;25:96. doi: 10.1186/s13058-023-01695-4 (PMC10426190; doi:10.1186/s13058-023-01695-4)
Supplement: Supplementary file 1 — Additional file 1: Supporting docking figures and pharmacological data in vitro and in vivo. [file 13058_2023_1695_MOESM1_ESM.docx]

**Supplementary figures:**


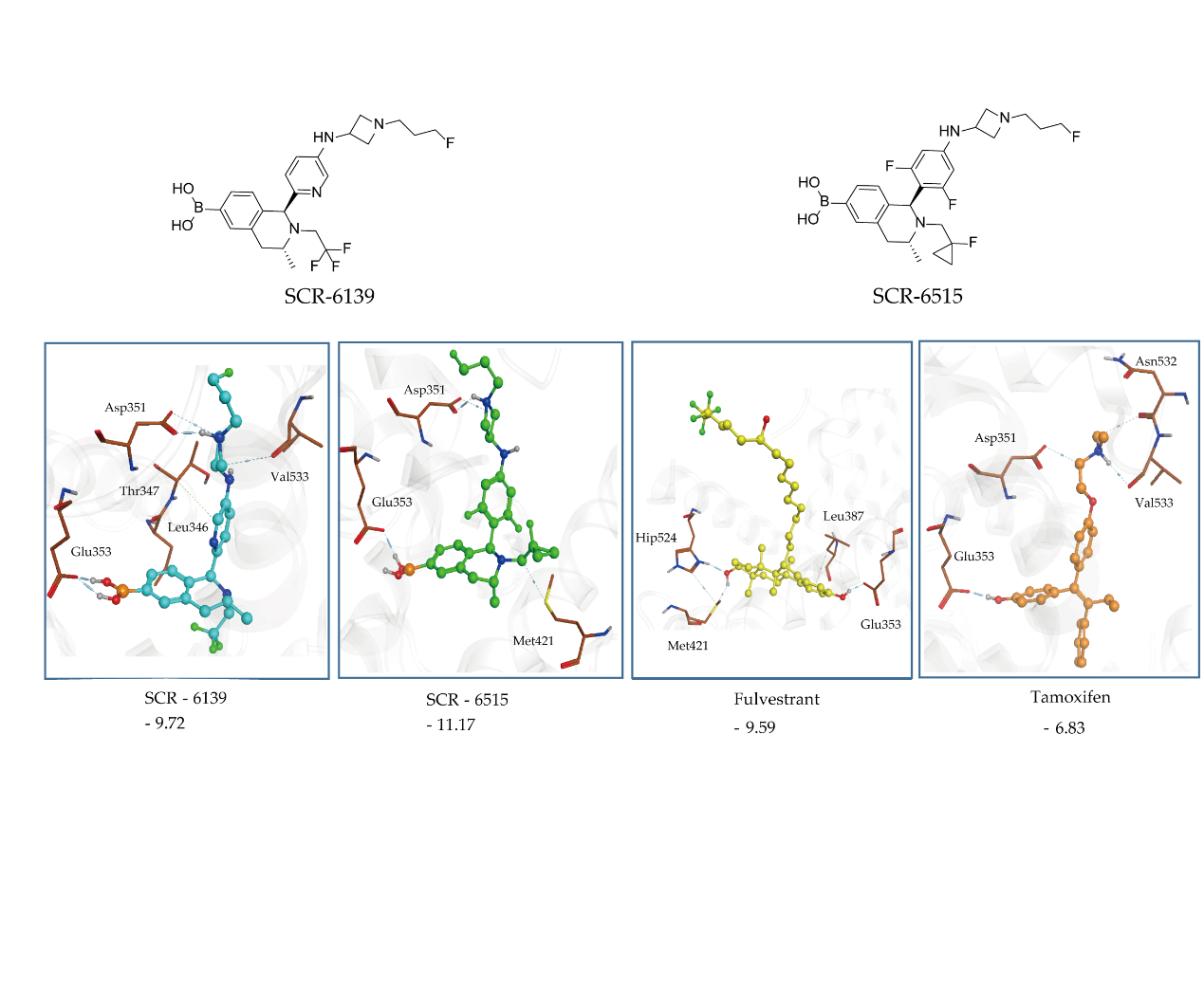


Figure S1: The structural formula of SCR-6139 and SCR-6515. In silico molecular modeling studies showed the superposition of SCR-6139 (blue), SCR-6515 (green), fulvestrant (yellow), and 4-hydroxy tamoxifen (orange) in the antagonistic binding pocket of ERα.


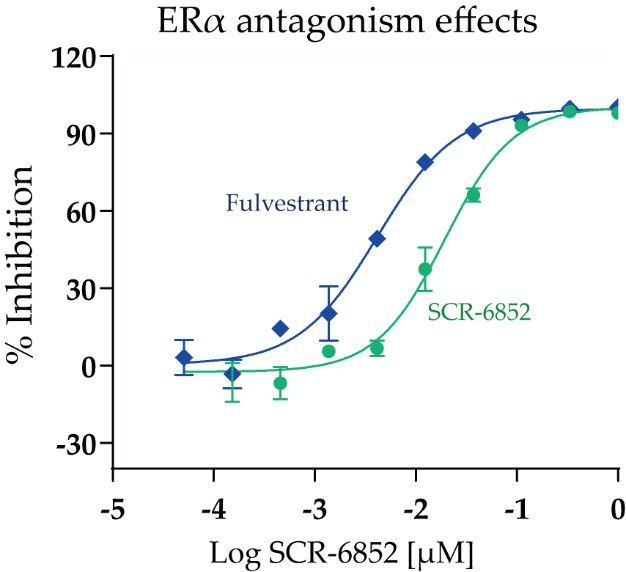


Figure S2: A nuclear translocation assay was used to evaluate the antagonistic activities of SCR-6852 or Fulvestrant. HEK293T cells were transfected with pBIND-ERα and pGL4.35 [luc2P/9XGAL4 UAS/Hygro] plasmids, followed by Estriol stimulation. After 24hr incubation, cells were then seeded into the 384-well plate and treated with linear-dilution compounds for 20hr. Steady-Glo™ Luciferase Assay Reagent (Promega) was added and relative luminescence units (RLU) were measured using an Envision Multilabel Reader (Perkin Elmer). 0% inhibition was normalized to untreated samples. IC50 and Imax were analyzed with Prism GraphPad.


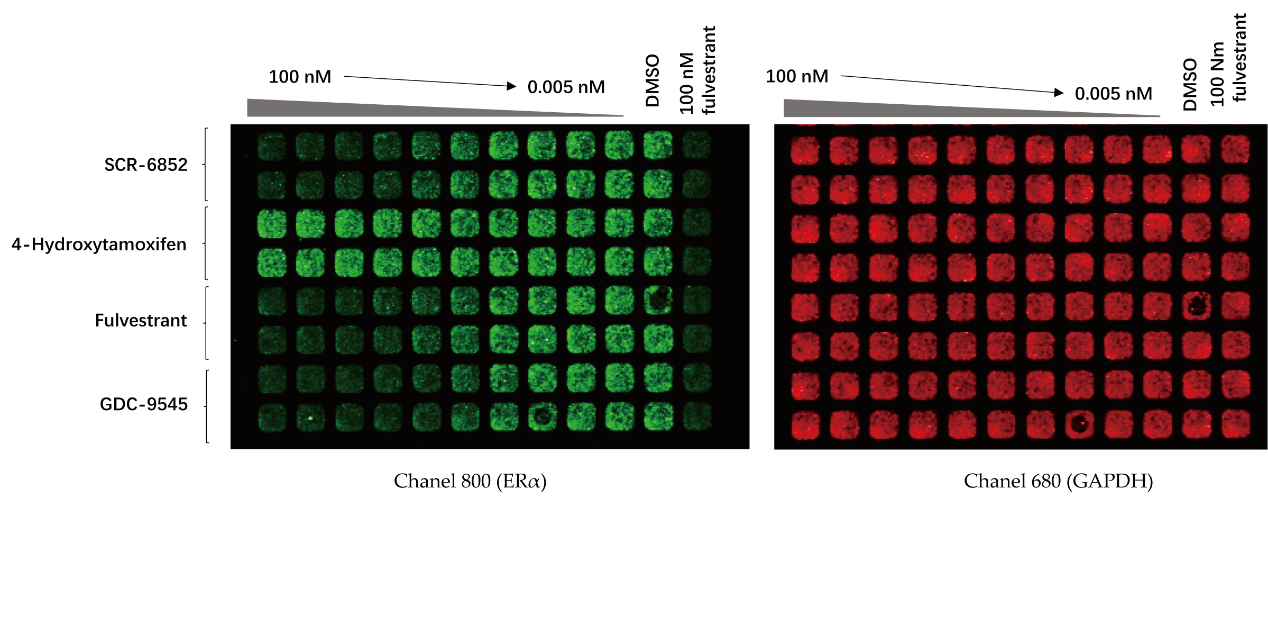


Figure S3: ERα degradation in MCF7 was determined using an In-Cell Western assay. Cells were seeded at a density of 5,000 cells per well into 384-well plates and treated with linear-titration compounds in duplicate for 24 hours. ERα levels were quantified by immunofluorescence assay as described in the method. The detection signals were captured by Odyssey.


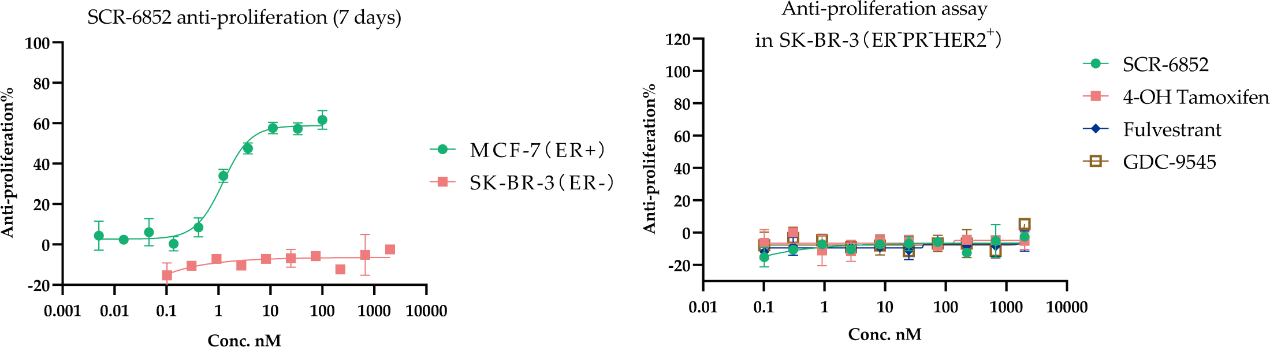


Figure S4: SCR-6852 exhibited selective inhibition activity on ER+ cell line proliferation compared to ER- cells. Cells were seeded in 384-well plates and treated with linear-dilution compounds for 7 days of incubation. Cell viability was assessed using CellTiter-Glo. Cell growth inhibition is presented as a percentage of CellTiterGlo activity relative to the vehicle control. 100% normalized to the culture medium.


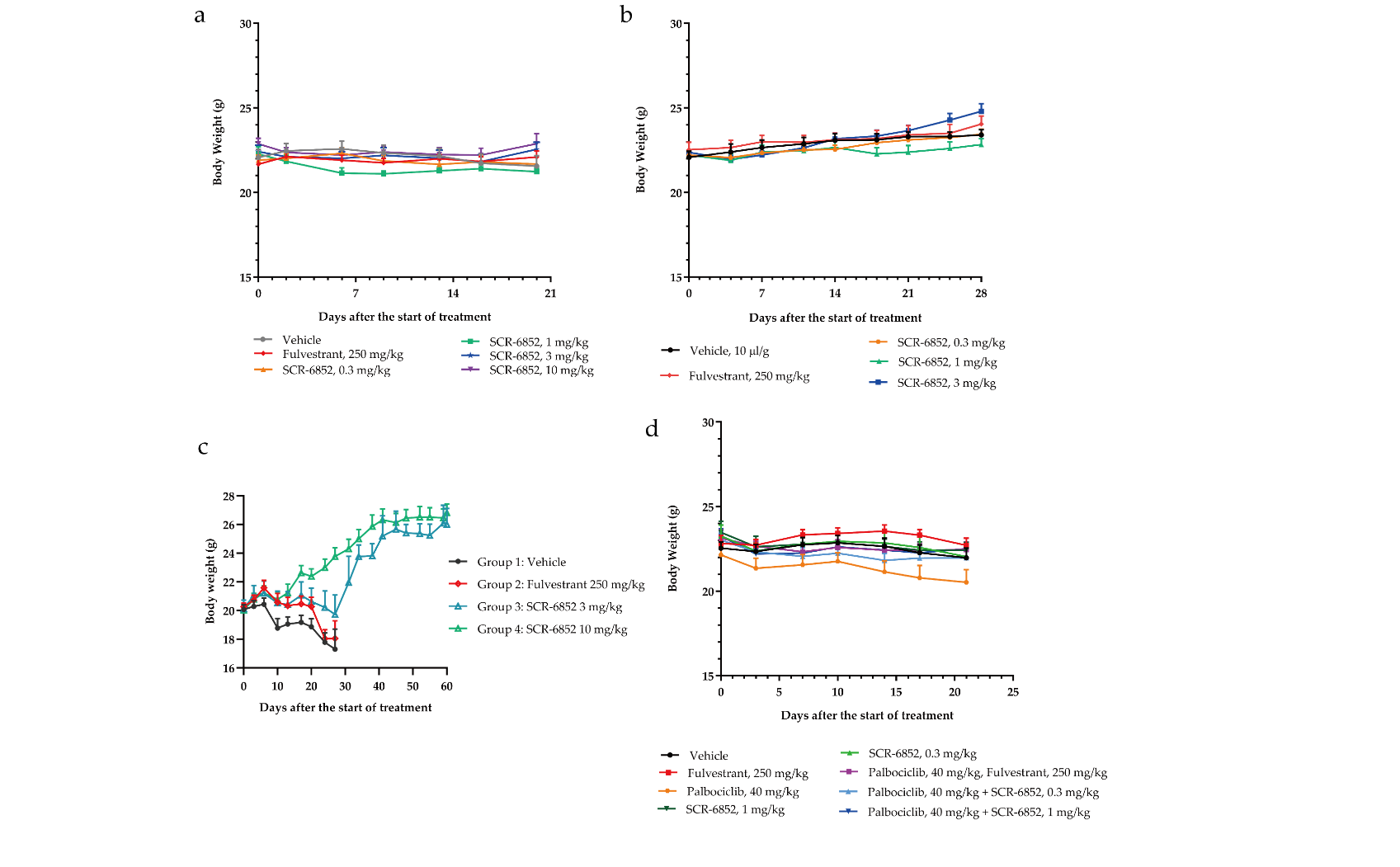
Figure S5: The *in vivo* anti-tumor studies were carried out in xenograft models. Balb/c nude mice or NPG mice with estrogen pellets were implanted with tumor cells subcutaneously or intracranially. Animals were treated with SCR-6852 (0.3, 1, 3, or 10 mg/kg, respectively, daily, orally) or 250 mg/kg fulvestrant (subcutaneous injection once a week). Tumor volume and body weights were evaluated twice per week until the study endpoint. No significant change was found during the treatments. a, MCF7 xenograft model; b, T47D model; c, the intracranial MCF-7 tumor model; d, MCF7 xenograft model.


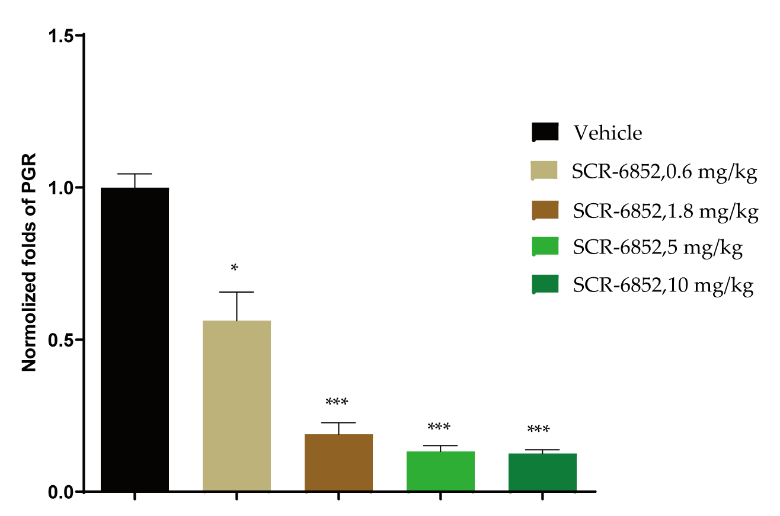


Figure S6: pharmacodynamic effect of SCR-6852 *in vivo.* Balb/c nude mice subcutaneously bearing estrogen pellets and MCF-7 tumors were treated with vehicle, 0.6 mg/kg, 1.8 mg/kg, 5 mg/kg or 10 mg/kg SCR-6852 by oral gavage once a day for 3 days with 3 mice per treatment group. Twenty-four hours after the final treatment, tumor tissues were snap-frozen by liquid nitrogen and RNA was extracted for each tumor tissue sample by RNeasy Mini Kit (Qiagen) according to the manufacturer’s protocol. RT-PCR for house-keeping gene GAPDH and Progesterone Receptor (PGR) were performed by High Capacity cDNA Reverse Transcription Kits (Applied Biosystems) and TaqMan® Gene Expression kit (Applied Biosystems). TaqMan probes: GAPDH, Cat. No. ABI-4331182, Assay ID Hs99999905_m1; PGR, Cat. No. ABI-4331182, Assay ID. Hs00172183_m1. The relative quantities were determined using ΔΔ threshold cycle (ΔΔCt).
