# Supplementary material for: SCR-6852, an oral and highly brain-penetrating estrogen receptor degrader (SERD), effectively shrinks tumors both in intracranial and subcutaneous ER + breast cancer models
Source: Breast Cancer Res. 2023 Aug 14;25:96. doi: 10.1186/s13058-023-01695-4 (PMC10426190; doi:10.1186/s13058-023-01695-4)
Supplement: Supplementary file 2 — Additional file 2: Target selectivity and transcription raw data. [file 13058_2023_1695_MOESM2_ESM.docx]

**Supplementary Table S1**

Table S1: Selectivity and *in vitro* safety of SCR-6852

| Target | SCR-6852, IC_50_ µM | Selectivity fold |
| --- | --- | --- |
| Estrogen Receptor alpha (ERα)**^※^** | 0.019 |  |
| Estrogen Receptor beta (ERβ) **^※^** | 0.104 | 5.5 |
| Progesterone Receptor**^※^** | 7.847 | 413 |
| Androgen Receptor**^※^** | >10 | >500 |
| Safety panel**^#^ (73 assays)** | >10 | >500 |
| Muscarinic acetylcholine receptor M 1 | 2.02 | 106 |
| Muscarinic acetylcholine receptor M 2 | 0.854 | 45 |
| 5-HT (HTR3A) | 5.15 | 271 |
| Nav1.5 | 6.33 | 333 |
| Dopamine transporter | 3.84 | 202 |

**^※^**Selectivity assays were performed using a nuclear translocation-antagonist assay. Safety panel, screened on SAFERYscan E/IC50 ELECT-78 assays, by Eurofins Discovery. Safety panel**^#^** data including targets except the following five (DAT, Nav1.5, 5-HT, CHRM1, CHRM2**).**

**Supplementary Table S2**

Table S2. FPKM of differential expression genes in MCF7

| Gene_ID | E2 @ 1 nM | SCR-6852 | Fulvestrant | 4-OHT |
| --- | --- | --- | --- | --- |
| TUBA1B | 1112.26 | 335.99 | 473.72 | 555.45 |
| TUBB | 568.06 | 174.85 | 230.78 | 291.22 |
| HMGB2 | 247.66 | 87.38 | 118.94 | 125.20 |
| PRSS23 | 215.68 | 48.61 | 49.30 | 68.31 |
| H2AX | 195.62 | 74.00 | 89.38 | 94.16 |
| MCM7 | 195.09 | 52.85 | 65.17 | 84.30 |
| CA2 | 184.51 | 96.74 | 87.89 | 157.09 |
| FKBP4 | 141.57 | 47.93 | 43.52 | 79.90 |
| PCNA | 139.06 | 41.40 | 48.34 | 59.31 |
| STC2 | 134.11 | 25.81 | 28.34 | 84.89 |
| IGFBP4 | 131.73 | 34.99 | 26.82 | 56.87 |
| TK1 | 126.85 | 37.60 | 42.16 | 57.95 |
| UBE2T | 108.61 | 31.24 | 45.52 | 41.94 |
| MYBL2 | 105.97 | 29.57 | 29.90 | 46.03 |
| LMNB1 | 103.66 | 41.69 | 49.03 | 53.86 |
| RRM2 | 98.16 | 15.20 | 24.81 | 33.29 |
| CKS1B | 97.65 | 36.52 | 46.61 | 49.15 |
| GFRA1 | 93.16 | 27.11 | 26.23 | 84.37 |
| GLA | 91.41 | 28.63 | 31.47 | 41.63 |
| MCM4 | 90.54 | 34.95 | 38.73 | 49.01 |
| MCM2 | 79.68 | 18.79 | 24.98 | 31.78 |
| ASF1B | 78.22 | 21.35 | 26.04 | 33.57 |
| TYMS | 74.96 | 19.81 | 25.13 | 31.45 |
| MCM3 | 73.54 | 24.28 | 30.18 | 34.54 |
| ATAD2 | 69.95 | 17.98 | 23.77 | 29.15 |
| TOP2A | 69.34 | 21.48 | 29.21 | 30.91 |
| FEN1 | 69.25 | 19.01 | 24.35 | 31.76 |
| UBE2C | 67.26 | 19.87 | 32.42 | 31.96 |
| DKK1 | 65.08 | 36.17 | 26.72 | 41.67 |
| PCLAF | 64.57 | 21.07 | 28.12 | 33.01 |
| UNG | 64.43 | 25.98 | 29.02 | 32.49 |
| RNASEH2A | 63.93 | 22.07 | 24.71 | 30.81 |
| GREB1 | 60.67 | 3.66 | 3.94 | 4.76 |
| PKIB | 60.35 | 18.64 | 16.14 | 26.05 |
| BRIP1 | 54.99 | 12.86 | 17.34 | 22.65 |
| CA12 | 52.56 | 18.98 | 18.70 | 46.02 |
| CDT1 | 51.94 | 16.57 | 21.42 | 21.06 |
| LIG1 | 50.95 | 16.64 | 19.47 | 23.58 |
| CCN5 | 50.43 | 16.55 | 22.35 | 29.67 |
| BIRC5 | 50.28 | 21.82 | 23.46 | 26.74 |
| MCM6 | 49.04 | 15.10 | 18.69 | 24.55 |
| FANCI | 48.65 | 11.61 | 16.43 | 20.35 |
| ANLN | 47.32 | 14.97 | 18.71 | 22.77 |
| MKI67 | 43.57 | 14.99 | 19.11 | 21.80 |
| AURKB | 43.03 | 12.49 | 19.26 | 18.53 |
| NCAPG2 | 42.44 | 11.83 | 15.05 | 19.25 |
| PKMYT1 | 41.08 | 7.25 | 9.90 | 12.25 |
| TACC3 | 40.66 | 15.23 | 19.06 | 21.35 |
| RRM1 | 40.40 | 13.36 | 19.08 | 20.41 |
| RFC2 | 36.71 | 12.27 | 14.47 | 18.64 |
| GINS2 | 36.18 | 6.45 | 8.09 | 12.84 |
| CCNA2 | 35.91 | 12.10 | 15.79 | 17.16 |
| CDCA4 | 35.30 | 8.69 | 12.74 | 15.28 |
| PBK | 34.81 | 8.68 | 14.24 | 16.47 |
| CDK1 | 34.56 | 9.72 | 14.26 | 15.34 |
| SLC6A14 | 33.54 | 8.40 | 8.71 | 24.37 |
| CDCA5 | 32.97 | 8.97 | 11.52 | 13.04 |
| KIFC1 | 32.81 | 10.64 | 12.36 | 15.12 |
| RECQL4 | 32.15 | 9.05 | 10.79 | 13.74 |
| SFXN2 | 31.80 | 6.10 | 7.17 | 8.23 |
| MCM5 | 31.11 | 9.05 | 9.71 | 13.27 |
| ADCY1 | 29.74 | 9.77 | 6.76 | 19.17 |
| PRIM1 | 28.80 | 6.61 | 7.88 | 10.94 |
| PAQR4 | 27.39 | 9.79 | 9.57 | 12.98 |
| E2F1 | 27.20 | 4.36 | 7.00 | 8.56 |
| RFC4 | 27.17 | 8.38 | 10.05 | 14.31 |
| UHRF1 | 26.62 | 4.72 | 6.71 | 9.20 |
| KIF11 | 25.57 | 8.84 | 11.20 | 13.08 |
| POLE | 25.41 | 9.02 | 11.56 | 12.83 |
| POLD1 | 24.97 | 9.77 | 11.19 | 11.86 |
| AGR3 | 24.59 | 1.98 | 2.46 | 25.86 |
| GINS1 | 23.84 | 7.73 | 8.52 | 11.19 |
| MELK | 23.52 | 7.20 | 9.66 | 11.34 |
| FANCA | 22.66 | 8.06 | 9.92 | 10.70 |
| CDC45 | 22.61 | 2.93 | 5.51 | 5.79 |
| TCOF1 | 21.81 | 9.05 | 9.75 | 10.86 |
| NPY1R | 21.66 | 3.51 | 3.20 | 8.44 |
| RAB31 | 21.61 | 7.68 | 6.58 | 11.89 |
| TONSL | 21.56 | 7.70 | 9.44 | 9.87 |
| TRIP13 | 21.48 | 6.56 | 6.92 | 8.37 |
| TCF19 | 21.19 | 5.35 | 6.28 | 8.96 |
| XRCC3 | 19.64 | 6.91 | 5.83 | 8.89 |
| TMEM164 | 18.93 | 6.30 | 6.22 | 10.65 |
| CXCL12 | 18.59 | 2.46 | 1.70 | 3.86 |
| BUB1B | 18.35 | 5.53 | 8.27 | 9.15 |
| ESPL1 | 17.48 | 5.59 | 7.73 | 7.94 |
| ORC6 | 17.34 | 5.43 | 5.57 | 7.62 |
| CCNE2 | 17.15 | 4.04 | 4.70 | 7.22 |
| SPC24 | 17.07 | 3.75 | 6.59 | 7.06 |
| ELOVL2 | 16.88 | 4.24 | 3.33 | 7.72 |
| NCAPG | 16.64 | 3.91 | 5.76 | 7.03 |
| SPC25 | 16.49 | 4.03 | 6.79 | 7.65 |
| CDC6 | 16.34 | 3.11 | 4.59 | 6.08 |
| ZNF367 | 15.85 | 2.36 | 4.18 | 5.84 |
| MAD2L1 | 15.82 | 5.09 | 6.32 | 7.76 |
| DSCC1 | 15.71 | 3.54 | 4.38 | 4.49 |
| KNTC1 | 15.58 | 4.71 | 5.81 | 6.86 |
| WDR62 | 15.52 | 4.50 | 5.55 | 5.88 |
| FANCD2 | 15.48 | 3.19 | 5.42 | 6.23 |
| KLK11 | 14.79 | 2.07 | 1.75 | 4.66 |
| SMC2 | 14.75 | 5.55 | 6.16 | 7.20 |
| MYB | 14.56 | 3.62 | 3.86 | 5.81 |
| POLA2 | 14.49 | 3.59 | 4.51 | 5.57 |
| SKA3 | 14.05 | 3.31 | 4.69 | 5.48 |
| RAD51AP1 | 13.82 | 3.16 | 5.24 | 5.44 |
| RAMP3 | 13.53 | 0.61 | 0.40 | 3.44 |
| C1orf226 | 13.05 | 4.50 | 3.74 | 5.19 |
| CENPU | 12.95 | 3.62 | 4.96 | 5.41 |
| CDCA3 | 12.59 | 4.44 | 6.06 | 6.21 |
| FAM111B | 12.41 | 1.32 | 2.82 | 4.32 |
| RAD54L | 12.35 | 2.57 | 3.44 | 4.39 |
| CAV1 | 11.53 | 6.76 | 5.48 | 5.56 |
| MCM10 | 11.30 | 1.85 | 2.51 | 3.64 |
| IGSF1 | 11.09 | 2.45 | 3.55 | 3.37 |
| TICRR | 10.96 | 2.67 | 3.71 | 4.37 |
| E2F8 | 10.32 | 2.27 | 3.66 | 4.23 |
| WDHD1 | 10.21 | 3.24 | 4.38 | 4.95 |
| EXO1 | 10.16 | 1.34 | 2.15 | 3.27 |
| RET | 10.12 | 2.63 | 1.74 | 4.49 |
| EME1 | 10.05 | 2.84 | 3.73 | 4.92 |
| RFC3 | 9.92 | 3.72 | 3.82 | 4.62 |
| DDIAS | 9.79 | 2.35 | 2.63 | 4.48 |
| ORC1 | 9.75 | 2.00 | 2.67 | 3.13 |
| RAD51 | 9.69 | 2.45 | 3.28 | 4.02 |
| E2F7 | 9.64 | 2.15 | 2.55 | 3.12 |
| KIF18B | 9.55 | 2.92 | 4.13 | 3.84 |
| BRCA1 | 9.45 | 2.12 | 2.96 | 3.58 |
| GPRIN1 | 8.93 | 3.61 | 2.95 | 4.22 |
| CD99-2 | 8.67 | 14.55 | 1.09 | 0.97 |
| RERG | 8.59 | 1.71 | 2.17 | 3.48 |
| CDCA7 | 8.46 | 1.38 | 1.84 | 2.77 |
| PGR | 8.19 | 0.52 | 0.52 | 1.93 |
| BLM | 8.02 | 1.56 | 2.45 | 3.14 |
| KNL1 | 7.74 | 2.43 | 3.41 | 4.05 |
| RAPGEFL1 | 7.70 | 2.67 | 2.18 | 6.63 |
| XRCC2 | 7.64 | 2.11 | 2.48 | 3.37 |
| MYBL1 | 7.63 | 1.00 | 0.96 | 1.32 |
| HELLS | 7.42 | 2.43 | 2.89 | 3.37 |
| ESCO2 | 7.26 | 1.35 | 2.24 | 2.98 |
| SHCBP1 | 7.21 | 2.15 | 2.87 | 3.43 |
| CDC25A | 7.21 | 1.60 | 1.67 | 2.63 |
| DNA2 | 7.03 | 2.47 | 2.77 | 3.39 |
| DTL | 6.96 | 1.11 | 1.38 | 2.21 |
| CALCR | 6.65 | 1.08 | 0.77 | 4.03 |
| CENPI | 6.53 | 2.18 | 2.93 | 3.21 |
| ASMTL-2 | 6.46 | 7.22 | 1.41 | 3.33 |
| POLQ | 6.41 | 2.06 | 2.64 | 2.74 |
| POLE2 | 6.15 | 1.78 | 2.45 | 2.61 |
| POLA1 | 6.08 | 2.26 | 2.69 | 3.09 |
| CLSPN | 5.95 | 1.19 | 1.80 | 1.96 |
| RBL1 | 5.83 | 1.72 | 2.06 | 2.35 |
| BRCA2 | 5.65 | 1.51 | 2.04 | 2.51 |
| E2F2 | 5.64 | 0.95 | 1.27 | 1.96 |
| CHEK1 | 5.59 | 1.53 | 2.32 | 2.63 |
| ASCL1 | 5.48 | 0.89 | 0.54 | 2.50 |
| WDR76 | 5.46 | 1.31 | 1.69 | 1.87 |
| HEY2 | 5.27 | 0.82 | 0.43 | 1.93 |
| CHAF1B | 5.21 | 1.51 | 1.52 | 2.31 |
| FIGNL1 | 5.08 | 1.67 | 2.36 | 2.46 |
| PDZK1 | 5.01 | 1.05 | 0.47 | 3.59 |
| COL12A1 | 4.88 | 1.96 | 1.73 | 3.75 |
| ERCC6L | 4.70 | 1.06 | 1.34 | 1.82 |
| TMEM26 | 4.29 | 1.27 | 0.84 | 2.14 |
| TP73 | 3.93 | 1.09 | 1.03 | 1.32 |
| ARHGAP36 | 3.84 | 0.54 | 0.28 | 1.42 |
| ACTG2 | 3.78 | 1.44 | 1.20 | 2.33 |
| LHX2 | 3.63 | 1.00 | 1.30 | 1.62 |
| ZGRF1 | 3.51 | 1.15 | 1.75 | 1.82 |
| AMZ1 | 3.24 | 1.13 | 0.77 | 3.21 |
| C5AR2 | 3.18 | 0.50 | 0.51 | 2.33 |
| GP1BB | 2.96 | 0.44 | 0.79 | 0.99 |
| GPR68 | 2.46 | 0.18 | 0.13 | 0.38 |
| FAM20A | 2.20 | 2.76 | 0.46 | 2.46 |
| HBB | 2.07 | 0.00 | 0.00 | 2.54 |
| CDH26 | 1.84 | 0.31 | 0.21 | 0.69 |
| TGM2 | 1.78 | 0.32 | 0.56 | 3.69 |
| DPYSL5 | 1.59 | 0.26 | 0.36 | 0.63 |
| RUBCNL | 1.40 | 0.16 | 0.10 | 1.01 |
| BMPER | 1.39 | 0.10 | 0.11 | 0.18 |
| ITGA2B | 1.22 | 0.18 | 0.30 | 0.44 |
| DHRSX-2 | 1.19 | 0.88 | 0.11 | 1.54 |
| GJA1 | 1.09 | 0.14 | 0.06 | 0.31 |
| SDK2 | 1.00 | 0.19 | 0.25 | 0.95 |
| ATRNL1 | 0.42 | 0.08 | 0.05 | 0.29 |
| GATA4 | 0.17 | 0.00 | 0.00 | 0.08 |
